# Meat and meat products – a scoping review for Nordic Nutrition Recommendations 2023

**DOI:** 10.29219/fnr.v68.10538

**Published:** 2024-02-21

**Authors:** Jelena Meinilä, Jyrki K. Virtanen

**Affiliations:** 1 Department of Food and Nutrition, University of Helsinki, Helsinki, Finland; 2 School of Medicine, Institute of Public Health and Clinical Nutrition, University of Eastern Finland, Kuopio, Finland

**Keywords:** meat, red meat, processed meat, poultry, dietary recommendations

## Abstract

Meat is not only a source of several nutrients but also a proposed risk factor for several non-communicable diseases. Here, we describe the totality of evidence for the role of meat intake for chronic disease outcomes, discuss potential mechanistic pathways, knowledge gaps, and limitations of the literature. Use of the scoping review is based on a *de novo* systematic review (SR) and meta-analysis on the association between poultry intake and cardiovascular disease (CVD) and type 2 diabetes (T2D), qualified SRs (as defined in the Nordic Nutrition Recommendations 2023 project) on meat intake and cancer by the World Cancer Research Fund (WCRF), the International Agency for Research on Cancer (IARC), and a systematic literature search of SRs and meta-analyses. The quality of the SRs was evaluated using a modified AMSTAR 2 tool, and the strength of evidence was evaluated based on a predefined criteria developed by the WCRF. The quality of the SRs was on average critically low. Our findings indicate that the evidence is too limited for conclusions for most of the chronic disease outcomes. However, findings from qualified SRs indicate strong evidence that processed meat increases the risk of colorectal cancer and probable evidence that red meat (unprocessed, processed, or both) increases the risk. The evidence suggests that both unprocessed red meat and processed meat (also including processed poultry meat) are probable risk factors for CVD mortality and stroke, and that total red meat and processed meat are risk factors for CHD. We found no sufficient evidence suggesting that unprocessed red meat, processed red meat, total red meat, or processed meat (including red and white meat) would be protective of any chronic disease. There was also no sufficient evidence to conclude on protective effect of poultry on any chronic diseases; effects on the risk of CVD, stroke, and T2D, to any direction, were regarded as unlikely.

## Popular scientific summary

- Pork, beef, and lamb are defined as red meat, while poultry (chicken and turkey) is considered white meat.
- Processed meat refers to red or white meat preserved by smoking, curing, or fermenting, or by the addition of salt and other preservatives.
- The total meat intake in the Nordic and Baltic countries ranges from about 100 to 200 g/day.
- Meat is a significant source of nutrients, such as protein, vitamins, minerals, and fatty acids, while processed meat is also a large source of salt.
- High intake of red and processed meat is associated with increased risk of colorectal cancer, coronary heart disease, stroke, and mortality from cardiovascular diseases.
- Evidence for an effect of poultry on the risk of chronic diseases is insufficient.

Meat commonly refers to ‘red meat’ from pork, beef, and lamb and to ‘white meat’ from chicken and turkey. In Western countries, red meat is a significant source of energy and several nutrients. Red meat is not only a good source of, for example, protein and essential amino acids; vitamins B_1_, B_2_, B_6_, and B_12_; iron; and zinc but also a notable source of unfavorable saturated fatty acids (SFA). High intake of red meat, unprocessed and processed, has been linked to a higher risk of several major chronic diseases, such as some cancers, cardiovascular diseases (CVDs), and type 2 diabetes (T2D). Some proposed mechanisms for this include the SFA and heme iron content in red meat. The increased risks have been especially observed with high intake of processed meat. Although meat is usually processed before it is consumed (by at least adding salt and baking or frying), ‘processed meat’ generally refers to a meat product that has been industrially processed by adding, for example, sodium, nitrites, or other preservatives or coloring agents, or by smoking, drying, curing, or fermenting. The added substances or substances formed during the meat processing (such as polycyclic aromatic hydrocarbons, advanced glycation end products, and heterocyclic aromatic amines) are among the factors that have been suggested to contribute to the increased disease risk from high processed meat intake. Absorption of iron is more efficient from meat compared to plant-based sources. This is because of the different form, heme iron, in meat compared to plant-based sources, and because of inhibitors of absorption in plant-based foods (1). On the other hand, high content of heme iron has been linked with adverse health outcomes, although the evidence is uncertain (2–4). Compared to red meat, poultry consumption is somewhat lower in Western countries (5), although the consumption has increased in recent years in most Nordic and Baltic countries (6). Poultry is a good source of protein and essential amino acids, and vitamins B_1_, B_2_, B_6_, and B_12_, and it contains less SFA compared to red meat. Less research data, compared to red and processed meat, exist on the association of poultry intake with health. The existing data indicate mainly no association or in some cases inverse association with disease risk.

The aim of this scoping review is to describe the totality of evidence for the role of meat intake for chronic disease outcomes as a basis for setting and updating the food-based dietary guidelines in the Nordic Nutrition Recommendations (NNR) 2023 (7) (Box 1).

Box 1 Background papers for Nordic Nutrition Recommendations 2023

- This paper is one of many scoping reviews commissioned as part of the Nordic Nutrition Recommendations 2023 (NNR2023) project (7)
- The papers are included in the extended NNR2023 report, but, for transparency, these scoping reviews are also published in Food & Nutrition Research
- The scoping reviews have been peer reviewed by independent experts in the research field according to the standard procedures of the journal
- The scoping reviews have also been subjected to public consultations (see report to be published by the NNR2023 project)
- The NNR2023 committee has served as the editorial board
- While these papers are a main fundament, the NNR2023 committee has the sole responsibility for setting dietary reference values in the NNR2023 project

## Methods

This scoping review follows the protocol developed within the NNR2023 project (7). The sources of evidence used in this review follow the eligibility criteria described previously (8).

The evidence for the associations of poultry consumption with CVD and T2D is based on a *de novo* systematic review (SR) commissioned by NNR2023 (7, 9). Qualified SRs on the association between meat and the majority of the most common cancer sites were available by the World Cancer Research Fund/American Institute of Cancer Research (WCRF/AICR) (red meat (unprocessed or combined unprocessed and processed depending on the cancer site), processed meat, and poultry) and by the International Agency for Research on Cancer (IARC) (red meat (unprocessed or combined unprocessed and processed depending on the cancer site) and processed meat) (3, 10, 11). The results of the associations between meat and cancer sites that WCRF reviewed in 2018 but did not find sufficient evidence to make conclusions are not included in the table of included studies (Table 1) but can be found from the webpages of WCRF (https://www.wcrf.org/diet-activity-and-cancer/risk-factors/meat-fish-dairy-and-cancer-risk/). The results of the associations between meat and those cancer sites for which WCRF has concluded on the strength of evidence are presented in Table 1 along with other included studies. The conclusions of IARC are reported in the text but not in the table because of the narrative nature of the IARC monograph.

**Table 1:** List of included 23 systematic reviews and meta-analyses (one SR can contribute to several outcomes) in setting the Nordic Nutrition Recommendations 2023 for the intake of meat and meat products

1st author (year), (reference)	Outcome(s)	Design of included primary studies	Definition of exposure	Exposure categories	Main findings (after RR or OR, 95% confidence interval in the parenthesis)	Evidence for heterogeneity (*I*^2^ >40% or *P* > 0.10)?Mentioned if heterogeneity explained	Evidence for publication bias?		AMSTAR-2 rating^†^
*Cardiovascular diseases*									
Zeraatkar et al. 2019 (19)	CVD mortality, CVD, fatal or non-fatal stroke, fatal or non-fatal MI	Prospective cohorts (No. of cases not reported) *N of studies/participants:* **Unprocessed red meat**: *Highest vs. lowest* CVD mortality: 8/389,528; CVD: 4/65,736; stroke: 6/102,024; fatal stroke: 3/268,504; MI: 1/55,171 *Reduction of 3 servings/wk* CVD mortality: 7/874,896; CVD: 3/191,803; stroke: 6/254,742; fatal stroke: 3/671,259; MI: 1/55,171 **Processed meat:** *Highest vs. lowest* CVD mortality: 9/>472,128 (the numbers in extreme categories unknown in some studies); CVD: 4/69,186; stroke: 6/101,861; fatal stroke: 2/231,992; MI: 1/55,171 *Reduction of 3 servings/wk* CVD mortality: 7/1,240,634; CVD: 3/200,421; stroke: 6/254,742; fatal stroke: 2/571,378 MI: 1/55,171 *Follow-up time in the original studies:* **Unprocessed red meat:** 5.5–28 **Processed meat:** 8–28	**Red meat**: mammalian meat; **Processed meat**: white or red meat preserved by smoking, curing, salting, or adding chemical compounds (for example, hot dogs, charcuterie, sausage, ham, and deli meats)	Reduction of 3 servings/wk Lowest vs. highest	**Unprocessed red meat** *Highest vs. lowest* CVD mortality: RR 0.88 (0.77–1.01); CVD: 0.92 (0.80–1.06); stroke: 0.90 (0.83–0.97); fatal stroke: 0.89 (0.76–1.05); MI: 0.85 (0.73–0.98) *Reduction of 3 servings/wk* CVD mortality: 0.90 (0.88–0.91); CVD: 0.95 (0.85–1.06); stroke: 0.94 (0.90–0.98); fatal stroke: 0.94 (0.89–0.99); MI: 0.93 (0.87–0.99) **Processed meat** *Highest vs. lowest* CVD mortality: 0.88 (0.77–1.00); CVD: 0.97 (0.88–1.05); stroke: 0.85 (0.80–0.93); fatal stroke: 0.92 (0.84–1.00); MI: 0.87 (0.79–0.95). *Reduction of 3 servings/wk* CVD mortality: 0.90 (0.84–0.97); CVD: 0.97 (0.87–1.09); stroke: 0.94 (0.90–0.98); fatal stroke: 0.95 (0.92–0.98); MI: 0.94 (0.91–0.98).	**Unprocessed red meat**: *Highest vs. lowest* Yes, CVD mortality: *I*^2^ = 83.4% (*P* = 0.001), also in studies with LoR*: *I*^2^ = 54.4% (*P* = 0.099) *Reduction of 3 servings/wk* Yes, CVD mortality: *I*^2^ = 86.9% (*P* < 0.01, HiR*). NA for MI **Processed meat:** *Highest vs. lowest* Yes, CVD mortality: *I*^2^ = 79.1% (*P* = 0.001), no heterogeneity in low risk of bias studies. *Reduction of 3 servings/wk* Yes, CVD mortality: *I*^2^ = 98.2% (*P* < 0.001, HiR); CVD: *I*^2^ = 75.1 (*P* < 0.01, LoR). NA for MI	Not done because < 10 studies		Moderate quality
Bechthold et al. 2019 (20)	Fatal or non-fatal CHD, fatal or non-fatal stroke, heart failure	Prospective cohorts *N of studies/cases* Highest vs. lowest comparisons: **Total red meat**: CHD: 3/6,659; stroke: 7/10,541; heart failure: 5/9,229 **Processed meat**: CHD: 5/7,038 cases; stroke: 6/9,492; heart failure: 3/7,077 *Follow-up time in the original studies:* **Total red meat:** 6–26 **Processed meat:** 4–26	**Total red meat (=processed and unprocessed red meat), processed meat**. No detailed definition.	Highest vs. lowest category Increase of 100 g/day of total red meat or 50 g/day of processed meat	**Total red meat** *Highest vs. lowest category* CHD: RR=1.16 (95% CI 1.08–1.24); stroke: 1.16 (1.08–1.25); heart failure: 1.12 (1.04–1.21) *per 100* g/day *increase* CHD: 1.15 (1.08–1.23); stroke: 1.12 (1.06–1.17); heart failure: 1.08 (1.02–1.12) Evidence for non-linear dose-response between red meat intake and heart failure (3 studies). **Processed meat** *highest vs. lowest category* CHD: 1.15 (0.99–1.33); stroke: 1.16 (1.07–1.26); heart failure: 1.27 (1.14–1.41) *Per 50* g/day *increase* CHD: 1.27 (1.09–1.49); stroke: 1.17 (1.02–1.34); heart failure: 1.12 (1.05–1.19). The association with stroke was stronger in US studies (1.47, 1.16–1.85) than in European studies (1.08, 0.96–1.22)	**Total red meat:** no **Processed meat**: yes, for stroke when assessed per 50 g/day higher intake (*I*^2^ = 56%, *P* = 0.05), mainly explained by the geographical location (USA vs. Europe)	Not done because < 10 studies		High quality
Cui et al. 2019 (21)	Heart failure	Prospective cohorts *No of studies/cases:* **Unprocessed red meat**: 5/8,281 **Processed meat:** 5/15,567 *Follow-up period:* **Unprocessed red meat**: 11.9–21.5 years. **Processed meat:** 8.2–21.5 years.	**Unprocessed red meat, processed meat.** No detailed definition.	Highest vs. lowest category	**Unprocessed red meat** RR=1.04 (95% CI 0.96–1.12) **Processed meat** 1.23 (1.07–1.41). In the stratified analyses, the association with increased risk was stronger in the European studies (1.33, 1.15–1.54) than in the US studies (1.08, 0.99–1.18).	**Unprocessed red meat:** no. **Processed meat:** yes (*I*^2^ = 58.9%, *P* = 0.05), which was mainly explained by the geographical location (USA vs Europe)	No		Critically low
Ramel et al. (9)	CVD incidence, CVD mortality, stroke incidence, stroke mortality *(only the results of meta-analysis are in the table. The other results are described in the text)*	Prospective cohorts N of studies/cases: CVD mortality: 6/1,158,411	**Processed and unprocessed white meat**: chicken, turkey	Highest vs. lowest category	Highest vs. lowest **CVD mortality:** RR: 0.95 (0.87–1.02)	CVD mortality: No I^2^ =25%, *P* = 0.16	Not done because of low number of studies		Qualified systematic review – No AMSTAR evaluation
*All-cause mortality*									
Zeraatkar et al. 2019 (19)	All-cause mortality	Prospective cohorts (No. of cases not reported) *No. of studies/participants:* **Unprocessed red meat:** *Reduction of 3 servings/wk* 8/893,436 *Highest vs. lowest* 9/413,760 **Processed meat:** *Reduction of 3 servings/wk* 8/1,241,900 *Highest vs. lowest* 10/>696,822 (some studies did not report the n in extreme categories) *Follow-up time:* **Unprocessed red meat:** 5.5–28 years. **Processed meat:** 9–28 years.	**Unprocessed red meat**: unprocessed mammalian meat; **Processed meat**: white or red meat preserved by smoking, curing, salting, or adding chemical compounds (for example, hot dogs, charcuterie, sausage, ham, and deli meats)	Reduction of 3 servings/wk Lowest vs. highest category	**Unprocessed red meat** *Reduction of 3 servings/wk* 0.93 (0.87–1.00) *Lowest vs. highest category* 0.90 (0.80–1.01) **Processed meat** *Reduction of 3 servings/wk* 0.92 (0.87–0.96) *Lowest vs. highest category* 0.88 (0.85–0.90)	**Unprocessed red meat:** *Reduction of 3 servings/wk* Yes, *I*^2^ = 96.0% (*P* < 0.001, 8 LoR studies) *Highest vs. lowest category* Yes, *I*^2^ = 94.6% (*P* < 0.001, 9 LoR studies) **Processed meat:** yes, *Reduction of 3 servings/wk* *I*^2^ = 86.0% (*P* < 0.001, 7 LoR studies) *Highest vs. lowest category* no	Not done because < 10 studies		Moderate quality
Schwingshackl et al. 2017 (31)	All-cause mortality	Prospective cohorts *No. of studies/cases:* **Total red meat:** *Highest vs. lowest category* 12/177,655 *Dose-response* 10/not reported **Processed meat**: *Highest vs. lowest category* 7/143,572 *Dose-response* 7/not reported *Follow-up time:* **Total red meat:** 5.5–28 years. **Processed meat:** 9–28 years.	**Total red meat, processed meat**. No detailed definition.	Highest vs. lowest category Increase of 100 g/day of total red meat or 50 g/day of processed meat	**Total red meat** *Highest vs. lowest category* RR=1.10 (95% CI 1.00–1.22) *Per 100 g/day increase* 1.10 (1.04–1.18) In stratified analyses, the positive association was observed mainly in studies with only men, with a longer follow-up, with a larger number of participants, with a validated dietary assessment method, and in the US vs. European or Asian studies. **Processed meat** *Highest vs. lowest category* 1.21 (1.16–1.26) *Per 50 g/day increases* 1.23 (1.12–1.36). In stratified analyses, the positive association was observed mainly in studies with a longer follow-up, with a larger number of participants, with a validated dietary assessment method, and in the US vs European studies.	**Total red meat:** yes *Highest vs. lowest category: I*^2^ = 93%, *P* < 0.001) *per 100 g/day increase: I*^2^ = 92%, *P* < 0.001). There was significant heterogeneity also in the stratified analyses (*I*^2^ = 56–95%). **Processed meat:** yes *Highest vs. lowest category: I*^2^ = 56%, *P* = 0.03) *Per 50 g/day increase: I*^2^ = 94%, *P* < 0.001). There was significant heterogeneity also in the stratified analyses (*I*^2^ = 85–95%), except for the studies conducted in Europe (*I*^2^ = 0%).	**Total red meat:** yes **Processed meat:** no		Critically low
*Hypertension*									
Zhang & Zhang 2018 (22)	Hypertension	Prospective cohorts *No. of studies/cases:* **Unprocessed red meat**: 5/23,854 **Processed red meat**: 5/23,854 **Poultry:** 6/14,739	**Unprocessed red meat**, **processed red meat, poultry.** No detailed definition.	Highest vs. lowest category	**Unprocessed red meat:** RR 1.19 (1.04–1.36) **Processed red meat:** RR 1.12 (1.02–1.23) **Poultry:** RR 1.15 (1.03–1.28)	**Unprocessed red meat:** yes, *I*^2^ = 91%, *P* < 0.001 **Processed red meat:** yes, *I*^2^ = 79.8%, *P* = 0.001 **Poultry:** yes, *I*^2^ = 63.3%, *P* = 0.02		No (for all meat types)	Critically low
Schwingshackl et al. 2017 (23)	Hypertension	Prospective cohort studies *No of studies/cases:* **Total red meat:** 7/97,745 **Processed meat:** 4/97,441 *Follow-up time:* **Total red meat**: 3–14 years and 564,247–1,396,062 person years **Processed meat**: 10–14 years and 564,247–1,396,062 person years	**Total red meat, processed meat**. No detailed definition.	**Total red meat:** Increase of 100 g/day **Processed meat:** Increase of 50 g/day	**Total red meat**: RR: 1.14 (1.02, 1.28); **Processed meat:** RR: 1.12 (1.00, 1.26)	**Total red meat:** yes, I^2^ = 88%, *P* < 0.001 **Processed meat:** yes, I^2^ = 82%, *P* < 0.001		Not done because <10 studies	Critically low
*Type 2 diabetes*									
Zeraatkar et al. 2019 (19)	T2D	Prospective cohorts (No. of cases not reported) *No of studies/participants* **Unprocessed red meat:** *Dose-response analyses:* 11/531,843 *Lowest vs. highest* 12/>211,467 (some studies did not report n in extreme categories) **Processed red meat:** *Dose-response analyses:* 17/758,540 *Lowest vs. highest* 19/>25,032 (some studies did not report n in extreme categories) *Follow-up time:* **Unprocessed red meat:** 4.6–28 **Processed red meat:** 4.3–28	**Unprocessed red meat**: mammalian meat; **Processed meat:** white or red meat preserved by smoking, curing, salting, or adding chemical compounds (for example, hot dogs, charcuterie, sausage, ham, and deli meats)	Reduction of 3 servings/wk Lowest vs. highest category	**Unprocessed red meat:** *Per 3 servings/wk reduction (1 serving equals 120 g)* RR 0.94 (95% CI 0.89–0.98) *Lowest vs. highest* RR 0.91 (0.84 to 0.98) Association stronger in low risk of bias studies **Processed meat:** *Per 3 servings/wk reduction (1 serving equals 50 g)* RR 0.85 (0.79–0.92), non-linear association *Lowest vs. highest* RR 0.83 (0.79–0.88) Association was weaker in low risk of bias studies	**Unprocessed red meat:** yes, *Per 3 servings/wk reduction* *I*^2^ = 64.9%, *P* < 0.01 No heterogeneity between studies with low risk of bias: *I*^2^ = 0.1%, *P* = 0.27 *Lowest vs. highest* *I*^2^ = 61.8%, *P* < 0.01 heterogeneity also in analysis including only low risk of bias studies (*I*^2^ = 64.8, *P* = 0.016) **Processed meat:** yes, *Per 3 servings/wk reduction* *I*^2^ = 92%, *P* < 0.001 heterogeneity also in analysis including only low risk of bias studies (*I*^2^ = 83.4, *P* < 0.001) *Lowest vs. highest* *I*^2^ = 56.9%, *P* < 0.01 heterogeneity also in analysis including only low risk of bias studies (*I*^2^ = 64.4, *P* = 0.004)		No (for all analyses)	Moderate quality
Schwingshackl et al. 2017 (23)	T2D	Prospective cohort studies *No. of studies cases:* **Total red meat:** 14/45,702 **Processed meat:** 14/43,781 *Follow-up time:* 4.6–28 years for both meat types	**Total red meat, processed meat.** No detailed definition.	**Total red meat**: 100 g/day increase; **Processed meat**: 50 g/day increase	**Total red meat** RR 1.17 (1.08–1.26) The results were unchanged in the subgroups of low risk of bias studies. The association was stronger in studies with longer follow-up (≥10 years), in the US, higher number of cases (≥1,000), and when outcome was self-reported or from registry (vs. diagnosed by physician). **Processed meat:** RR 1.37 (1.22–1.55) Evidence of a non-linear dose-response association; the risk of T2D increased by 30% with increasing intakes up to 50 g/day. Moderate additional detrimental effects for increasing intake above this value were observed. The association was stronger in low risk of bias studies, studies with women, individuals above 50 years, in the US, in studies with FFQ as a dietary intake method, and in studies with self-reported outcome measures	**Total red meat:** yes, I^2^ = 83%, *P* < 0.001 Heterogeneity persisted in stratified analyses. **Processed meat:** yes, *I*^2^ = 88%, *P* < 0.001 The observed heterogeneity persisted in stratified analyses	**Total red meat**: No **Processed meat:** Yes. Visual inspection of the funnel plot suggests that small studies showing inverse or null association may be missing		Critically low
Ramel et al. (9)	T2D	Prospective cohort studies *No. of studies/no. of participants:* 7/388,283	**Processed and unprocessed white meat**: chicken, turkey	Highest vs. lowest	RR 0.98 (0.87–1.11)	Yes, *I*^2^ = 82%, *P* = 0.81	Not done because of low number of studies		Qualified systematic review – No AMSTAR evaluation
*Cancers*									
WCRF 2018 (2, 3)	Colorectal cancer	Cohort, nested case-control and case-cohort designs *No. of studies/cases* **Unprocessed red meat:** 8/6,662 **Processed meat:** 10/10,738 **Poultry**: 6/3,429 *Follow-up time:* **Total red meat**: 6–24 years and 105,044–2,279,075 person years **Processed meat:** 3.3–24 years and 105,044–2,279,075 person years **Poultry:** 3.3–16 years and 105,044–286,731 person years	**Unprocessed red meat;** **Processed meat:** generally described as processed meat, preserved meat or cured meat, but individual items included in the meat group could vary between the studies; **Poultry**: chicken, turkey, ground poultry, and the processed poultry components of turkey or chicken cold cuts and low-fat versions of hot dogs and sausage	**Unprocessed red meat:** 100 g increase **Processed meat:** 50 g increase **Poultry:** 100 g	**Unprocessed red meat:** RR 1.12 (1.00–1.25) **Processed meat:** RR1.16 (1.08–1.26) **Poultry:** RR 0.81(0.53–1.25) From individual studies, only one study observed a significant inverse association.	**Unprocessed red meat:** no **Processed meat:** no **Poultry:** yes, *I*^2^ = 48.0%, *P* = 0.05	No (for all meat types)		Qualified systematic review – No AMSTAR evaluation
WCRF 2018 (2, 3) based on systematic review by Li et al. 2016 (27)	Nasopharyngeal cancer	Case-control studies *No. of studies/cases:* **Total red meat:** 6/911; **Processed meat:** 10/3,154 Follow-up time not reported.	**Total red meat, processed meat.** No detailed definition.	**Total red meat:** < 100 g/week vs. never, 100–300 g/week vs. never, > 300 g/week vs. never **Processed meat:** < 30 g/week vs. never, 30–60 g/week vs. never, > 60 g/week vs. never	**Total red meat:** *< 100 g/week vs. never* RR 1.35 (95% CI = 1.21–1.51) *100–300 g/week vs. never* RR 1.54 (95% CI = 1.35–1.76) *> 300 g/week vs. never* RR 1.71 (95% CI = 1.14–2.55) **Processed meat:** *< 30 g/week vs. never* RR 1.46 (95% CI = 1.31–1.64) *30–60 g/week vs. never* RR 1.59 (95% CI = 1.33–1.90) *> 60 g/week vs. never* RR 2.11 (95% CI = 1.31–3.42)	**Total red meat:** *< 100 g/week vs. never* No *100–300 g/week vs. never*: yes, *I*^2^ = 57%, *P* = 0.05 *> 300 g/week vs. never*: yes, *I*^2^ = 77%, *P* = 0.01 **Processed meat:** *< 30 g/week vs. never***:** yes, *I*^2^ = 76%, *P* < 0.01 *30–60 g/week vs. never***:** yes, *I*^2^ = 82%, *P* < 0.01 *> 60 g/week vs. never***:** yes, *I*^2^ = 85%, *P* < 0.01	No (for all analyses)		Qualified systematic review – No AMSTAR evaluation
WCRF 2018 (2, 3) (based on analysis from 2016 because no relevant new studies were found during the continuous updating processed in 2018)	Lung cancer	Cohort, nested case-control and case-cohort *No. of studies/cases* **Total red meat:** 7/9,765 **Processed meat:** 7/10,292 **Poultry:** 6/11,707 *Follow-up time:* 7–11.4 years for Total red meat, and for processed meat Poultry: 9.1–11.4 years	**Total red meat**, **processed meat, poultry**. No detailed definition.	**Total red meat:** 100 g/day increase **Processed meat:** 50 g/day **Poultry:** 100 g/day	**Total red meat:** RR 1.22 (1.02–1.46) **Processed meat:** RR 1.14 (1.05–1.24) **Poultry:** RR 0.91 (0.85–0.97) Only one study showed a significant inverse association	**Total red meat:** yes, *I*^2^ = 66%, *P* < 0.01 Heterogeneity probably explained by two studies that reported stronger associations than the average (concluded from funnel plot). **Processed meat:** no **Poultry:** no	**Total red meat:** No **Processed meat:** Yes (*P* = 0.04) **Poultry:** No		Qualified systematic review – No AMSTAR evaluation
WCRF 2018 (2, 3) (based on analysis from 2011 because no relevant new studies were found during the continuous updating processed in 2018)	Pancreatic cancer (combined incidence and mortality)	Prospective cohort studies *No. of studies/cases* **Total red meat:** 8/2,761 **Processed meat:** 6/2,748 *Follow-up time:* (reported only for studies found for the update of 2011) Processed meat: 5–13.3 Red meat: 5–16.3	**Total red meat,** **Processed meat.** No detailed definition.	**Total red meat:** 100 g/day **Processed meat:** 50 g /d increase	**Total red meat** RR 1.19 (0.98–1.45) In the analysis stratified by sex Men RR 1.43 (1.10–1.86, 3 studies) Women RR = 1.06 (0.86–1.31, 4 studies) **Processed meat** RR 1.17 (1.91–1.34) In subgroup analyses by sex, a positive association of processed meat was found in men, but not in women. Study results in women were inconsistent, showing associations to both directions.	**Total red meat:** yes, *I*^2^ = 52%, *P* = 0.04 Heterogeneity decreased in subgroups of men and women: Men (3 studies): no Women (4 studies): no **Processed meat:** no	No. for both meat types		Qualified systematic review – No AMSTAR evaluation
WCRF 2018 (2, 3)	Esophageal cancer	Cohort, nested case-control and case-cohort *No. of studies/cases* Mixed red meat and processed meat: 3/1,234 Processed meat: 4/1,388 No. of cases not reported for beef, lamb, pork, or poultry *No. of studies* Beef: 2 Pork: 3 Lamb: 1 Poultry: 3 *Follow-up time*: Red and processed meat: 6.8–9.7 years Processed meat: 6.8–25 years Not reported for other meat types.	**Mixed red meat and processed meat, processed meat**. Processed meat definitions in the primary studies: ham, sausages, bacon, sausages, processed meat, processed meat and fish; **Beef, pork, lamb, poultry**	**Mixed red meat and processed meat** 100 g/day **Processed meat** 50 g /d **Beef, pork, lamb, poultry** No meta-analysis	**Mixed red meat and processed meat** RR 1.22 (0.95–1.56) **Processed meat** RR 1.39 (1.09–1.77) association significant only in a subgroup of European studies. Association was not significant when only those studies were analysed that adjusted for alcohol and physical activity. **Beef:** no association (12) and borderline significant association (13) **Pork:** Borderline significant association in men (12); no association (13), significant positive association (14) **Lamb**: No association (13) **Poultry:** No association in analyses comparing the highest vs. lowest analysis intake categories (12, 15, 16)	Mixed red meat and processed meat, and processed meat: no Beef, pork, lamb, poultry: NA because no meta-analysis	Not assessed because <10 studies Beef, pork, lamb, poultry: NA		Qualified systematic review – No AMSTAR evaluation
WCRF 2018 (2, 3)	Stomach cancer	Cohort, nested case-control and case-cohort designs *No. of studies/cases* **Unprocessed red meat:** 4/2,408; **Processed meat:** 10/4,728; **Poultry:** 5/3,708 *Follow-up time:* **Total red meat**: 6.5–19 years **Processed meat**: 6.3–21 years **Poultry**: 6.5–19 years	**Unprocessed red meat, processed meat, mixed red and processed meat, poultry.** No detailed definition.	**Unprocessed red meat:** 100 g/day **Processed meat:** 50 g/day **Poultry:** 100 g/day	**Unprocessed red meat:** RR 1.12 (0.95–1.32) **Processed meat:** RR 1.19 (1.06–1.34), In a stratified analysis by geographical location, the result was significant only in subgroup of European studies. The results not significant when only those included that adjusted for physical activity. **Poultry:** RR 0.98 (0.82–1.17)	No (for all meat types)	**Unprocessed red meat:** NA **Processed meat:** Yes. 0.05 Asymmetry mainly driven by a small study that reported a very strong positive association. **Poultry** No		Qualified systematic review – No AMSTAR evaluation
Liu & Lin 2014 (28)	Thyroid cancer	Case-control studies *No. of studies/cases:* 5/831 *Follow-up time:* 2–9 years	**Total meat**	Highest vs. lowest category	OR 0.96 (0.70–1.34, 5 studies)	No	No		Critically low
Han et al. 2019 (29)	Total cancer mortality and incidence	Prospective cohort studies (No. of cases not reported) *No. of participants:* **Unprocessed red meat**: cancer mortality: 3/875,290 cancer incidence: 2/71,858 **Processed red meat** cancer mortality: 6/1,198,234 cancer incidence: 2/71,858 *Follow-up time:* Unprocessed red meat: on a range 5–28 years **Processed meat**: range 5–28 years	**Red meat:** meat from mammals **Processed meat**: meat that has been preserved by smoking, curing, salting, or adding preservatives (for example, hot dogs, charcuterie, sausage, ham, and cold-cut deli meats)	3 serving/wk reduction	**Unprocessed red meat** Cancer mortality: RR 0.93 (0.91–0.94) The result was similar in low-risk of bias studies with no heterogeneity. The result significant in only 1 primary study *Cancer incidence:* RR 0.93 (0.83–1.04) The results was significant in the 1 low-risk of bias study	**Unprocessed red meat** *Cancer mortality*: no *Cancer incidence:* yes, *I*^2^ = 50.9%, *P* = 0.15 **Processed meat:** *Cancer mortality*: yes, *I*^2^ = 53.9%, *P* = 0.04 *Cancer incidence*: yes, *I*^2^ = 69.7%, *P* = 0.07	Not done because no. of studies <10		Critically low
					**Processed meat** *Cancer mortality:* RR 0.93 (0.90–0.96), The result was similar in subgroup of low-risk of bias studies (3) with no evidence of heterogeneity. The result significant in only 1 primary study *Cancer incidence:* RR 0.99 (0.89–1.09) The result was similar in the one low risk of bias study.				
Zhang et al. 2018 (30)	Total cancer mortality	Prospective cohort studies *No. of cases in original studies* 257–9,861 *No. of studies* Highest vs. lowest: 8 Dose-response: 5 *Follow-up time* 5.5–22 years	**Poultry**	Highest vs. lowest category; 100 g/day	*Highest vs. lowest* RR 0.96 (0.93–1.00). Subgroup analysis showed a statistically significant inverse association in the subgroups of Asian studies, studies with shorter follow-up duration, high-quality studies and studies with large number of participants *Per 100 g/day increase* RR 0.97 (0.88–1.07)	No (for both analyses)	No (for both analyses)		Critically low
*Obesity*									
Schlesinger et al. 2019 (32)	Obesity	Prospective cohort studies *No. of studies/cases* 1/7,183 *Follow-up time:* Total red meat: 1–16 years	**Unprocessed and processed red meat**: Pork, veal, lamb, beef, mutton, processed red meat (sausages, salami, ham), hamburger, meatloaf; processed meat: Salami, cold-cut sausage, ham, fried sausage, liver sausage.	Highest vs. lowest category	**Total red meat:** No meta-analysis. Result of the individual study: RR 1.23 (1.07-1.41)	NA because no meta-analysis	NA		Not assessed
	Abdominal obesity	Prospective cohort studies *No. of studies/cases:* **Total red meat:** 2/1,500 **Processed meat:** 1/36 *Follow-up time:* **Total red meat**: 1->10 years **Processed meat**: 1 years	**Red meat**: Pork, veal, lamb, beef, mutton, processed red meat (sausages, salami, ham), hamburger, meatloaf; **Processed meat**: Salami, cold-cut sausage, ham, fried sausage, liver sausage.	**Total red meat** Highest vs. lowest category Increase of 100 g/day **Processed meat:** Highest vs. lowest category	**Total red meat** *Highest vs. lowest* RR 1.18 (95% CI: 1.06, 1.32) *Increase of 100 g/day* 1.10 (1.04, 1.16) **Processed meat** No meta-analysis. Result of an individual study RR 8.80 (95% CI: 1.20–64.28)	**Total red meat:** no **Processed meat:** NA because no meta-analysis	NA		
*Mental health*									
Zhang et al. 2017 (33)	Depression	Prospective cohort studies (N of cases not reported) *N of studies / participants* 3 / 20,072 *Follow-up time not reported*.	**Total meat**	Highest vs. lowest	RR = 1.13, 95% CI: 1.03 to 1.24)	no	No		Not assessed
*Other health outcomes*									
Schoenaker et al. 2016 (38)	Gestational diabetes	Prospective cohort study *No. of studies/cases:* 1/870 *Follow-up time* 10 years	**Total unprocessed, processed red meat**: beef, lamb, pork, hamburger, bacon, beef hot dogs and sausages, salami, and bologna	1 serving /d increase	No meta-analysis. Individual study: **Total red meat:** RR 2.05 (1.55–2.73), **Unprocessed red meat**: 1.60 (1.21–2.12) **Processed red meat:** 1.36 (1.03–1.80)	NA because no meta-analysis	NA		Not assessed
van Westing et al. 2020 (37)	Chronic kidney disease	Prospective cohort study (79) *No. of studies/cases:* 1/2,632 *Follow-up time:* 23 years	**Red meat (not clear whether unprocessed or total),** **processed meat,** **poultry.** No detailed definition.	Q5 vs. Q1	**Red meat (not clear whether unprocessed or total)** HR 1.19 (1.03–1.36) **Processed meat** HR 1.12 (0.98–1.29) **Poultry** HR 0.94 (0.84–1.06)	NA because no meta-analysis	NA		Not assessed
		Prospective cohort study (78) *No. of studies/cases:* 1/613 *Follow-up time:* 3.12 years	**Total red meat, processed red meat.** No detailed definition.	Q4 vs. Q1	**Total red meat** OR 1.73 (1.33–2.24) **Processed red meat** OR 1.99 (1.54–2.56)	NA because no meta-analysis	NA		
Guo et al. 2021 (34)	Metabolic syndrome	Prospective cohorts (No. of cases not reported) *No. of studies/participants:* **Total red meat**: 8/16,121 **Unprocessed red meat**: 3/5,535 **Processed red meat**: 4/5,959 **Poultry:** 3/7,270 *Follow-up time:* **Unprocessed red meat**: 1–6 years **Processed red meat**: 1–6 years	**Red meat:** beef, pork, horse, veal, deer, and lamb. **Processed red meat:** red meat products with ingredients (sausages, cold cuts, and others)	Highest vs. lowest category	**Total red meat:** 1.35 (1.13–1.62) **Unprocessed red meat:** 1.32 (1.14–1.54) **Processed red meat:** 1.48 (1.11–1.97) **Poultry:** 0.85 (0.75, 0.97)	**Total red meat**: yes, *I*^2^ = 54.4%, *P* = 0.03) No heterogeneity between studies with only non-Asian populations (4 studies) and between studies adjusting for physical activity (5 studies) **Unprocessed red meat:** no **Processed red meat**: yes, *I*^2^ = 64.7%; *P* = 0.04) **Poultry:** no	No		Not assessed
Salari-Moghaddam et al. 2018 (35)	Chronic obstructive pulmonary disease (COPD)	Prospective cohorts *No. of studies/cases:* Highest vs. lowest category: 5/8,338 Dose-response: 5/8,338 *Follow-up time:* 11.6–17 years	**Processed red meat:** sausages, cold cuts, ham, salami, blood pudding, liver pate, cured meat, bacon, hot dogs, bologna	Highest vs. lowest category Increase of 50 g/wk	*Highest vs. lowest category* HR = 1.40 (95% CI 1.21–1.62) *Increase of 50 g/wk* 1.08 (1.03–1.13)	*Highest vs. lowest category:* No *Increase of 50 g/wk:* yes, *I*^2^ = 90.6%, *P* < 0.001)	No		Not assessed
Li et al. 2018 (36)	Gout	Prospective cohorts *No. of studies/cases:* 2/2,897 Follow-up time: 11–12 years	**Red meat.** No detailed definition.	Highest vs. lowest category	Gout: OR=1.29 (95% CI 1.16–1.44)	No	No		Not assessed
Zeraatkar et al. 2019 (19), based on one primary study (80)	Anemia	Prospective cohort study *No. studies/cases:* 1/3,979 *Follow-up*: 3 years	**Red meat (unclear whether unprocessed or total red meat)**	Incident anemia (defined as anemia developed from at 3 years from the baseline) vs. no anemia Persistent anemia (defined as anemia detected at baseline and at 3 years) vs. no anemia	Incident anemia: OR 0.98 (0.90–1.06) Persistent anemia: OR 0.89 (0.79–1.01) At baseline mean intake of red meat 0.6 servings/d with no differences between those with anemia and those without anemia	NA because no meta-analysis.	NA		Not assessed

NA, not applicable.

* In this study, heterogeneity was investigated separately for high-risk (HR) and low-risk (LR) studies. Risk was based on Clinical Advances through Research and Information Translation (CLARITY) risk-of-bias instrument for cohort studies.

† One weakness in the critical domains of the AMSTAR-2 tool led to rating of ‘low’ and 2 or more weaknesses in the critical domains led to rating ‘critically low’.

To review the association between meat intake and other health outcomes, we performed a literature search on 13 September 2021 in PubMed and 29 October 2021 in Web of Science. The search string for PubMed search was (meat[MeSH Terms] OR meats[MeSH Terms]) AND (“2011”[Date - Publication] : “3000”[Date - Publication]) AND humans[Filter] AND (systematic review[Publication Type] OR meta-analysis[Publication Type]). The search string for Web of Science search was “(ALL=((meat OR meats OR beef OR lamb OR mutton OR pork OR poultry))) AND ALL=(systematic review OR meta-analysis) and Review Articles (Document Types)” in the following Web of Science categories: Respiratory System or Allergy or Gerontology or Integrative Complementary Medicine or Geriatrics Gerontology or Pediatrics or Behavioral Sciences or Obstetrics Gynecology or Clinical Neurology or Neurosciences or Rheumatology or Hematology or Peripheral Vascular Disease or Immunology or Orthopedics or Medicine Research Experimental or Surgery or Psychiatry or Cardiac Cardiovascular Systems or Gastroenterology Hepatology or Endocrinology Metabolism or Oncology or Medicine General Internal or Nutrition Dietetics. Additional relevant articles were found in PubMed. ‘Similar articles’ list and reference lists of umbrella SRs found in the PubMed and Web of Science searches.

Altogether 716 SRs were retrieved, whose titles and abstracts were reviewed for relevance. Altogether 153 SRs on meat intake and health outcomes were found. From the 153, the most recent and highest quality articles on each of the outcomes were referred to in this scoping review (*n* = 25, Table 1). Additionally, four of the SR:s were included in the section Mechanisms because they studied intermediate outcomes, for example, inflammation markers or blood lipids, but not disease outcomes. Articles not included in the review are described in Supplementary Table 1 (*n* = 124).

The quality of the SRs included in the review and derived from the literature search was evaluated using a modified AMSTAR 2-NNR tool (7, 17). The criteria for the ratings were as follows (YES = meets the criteria, NO = does not meet the criteria): High confidence: all critical domains YES, ≤ 2 non-critical domains NO; Moderate confidence: all critical domains YES, ≥ 3 non-critical domains NO; Low confidence: 1 critical domain NO, ≤ 2 non-critical domains NO; Critically low: ≥ 2 or more critical domains NO independent of non-critical domains, OR 1 critical domains NO and > 2 non-critical domains NO.

The critical domains of the tool concerned protocol registration, comprehensiveness of literature search, adequacy of risk of bias assessment, appropriate statistical methods, accounting risk of bias in interpretation of the results, and investigation of publication bias. The strength of evidence per outcome (with positive or negative association with meat intake) was evaluated based on predefined criteria developed by WCRF described by Arnesen et al. (18). The strength of evidence was not evaluated when there was no association between meat intake and an outcome, but it was reported if a qualified SR or the *de novo* SR included evaluation of such associations. A summary of the strength of evidence evaluations is presented in Table 2.

**Table 2:** Strength of evidence per meat type and per chronic disease outcome*

Chronic disease outcome	Unprocessed red meat	Processed red meat	Total red meat	Processed meat (incl. red and white meat)	Poultry
Total mortality	Limited – Suggestive ↑		Limited – Suggestive ↑	Limited – Suggestive ↑	
Cardiovascular disease	Limited – No conclusion ↑			Limited – No conclusion ↑	Limited – No conclusion
Cardiovascular disease mortality	Probable ↑			Probable ↑	Probable: no effect
Coronary heart disease			Probable ↑	Probable ↑	Limited – No conclusion
Stroke	Probable ↑		Probable ↑	Probable ↑	Limited – No conclusion
Stroke mortality	Limited-No conclusion ↑			Limited – No conclusion ↑	Probable: no effect
Myocardial infarction	Limited – No conclusion ↑			Limited – No conclusion ↑	
Heart failure			Limited – Suggestive ↑	Limited – Suggestive ↑	
Hypertension	Limited – No conclusion ↑	Limited – No conclusion ↑	Limited – No conclusion ↑	Limited – No conclusion ↑	Limited – No conclusion ↑
Type 2 diabetes	Limited – No conclusion ↑			Limited – Suggestive ↑	Probable: no effect
Total cancer incidence and mortality	Limited – No conclusion ↑				Limited – No conclusion
Colorectal cancer	Probable ↑			Convincing ↑	
Lung cancer			Limited – Suggestive ↑	Limited – Suggestive ↑	Limited – No conclusion ↓
Nasopharyngeal			Limited – Suggestive ↑	Limited – Suggestive ↑	
Pancreatic			Limited – Suggestive ↑	Limited – Suggestive ↑	
Esophageal				Limited – Suggestive ↑	
Stomach				Limited – Suggestive ↑	Limited – No conclusion

The upward pointing arrows refer to evidence of increased risk by increased intake, whereas the downward pointing arrows refer to evidence of decreased risk by increased intake. Lack of arrow refers to evidence of a lack of association. Evaluated based on the criteria of the World Cancer Research Fund.

* For outcomes that do not appear in the table and for the empty cells in the table, strength of evidence has not been evaluated because of no association between the meat type and the outcome or limited number or complete lack of studies. The strength of evidence for the lack of association was not evaluated, but those evaluated by Ramel et al. (Food & Nutrition Research, 2023) have been included (for associations between poultry and cardiovascular disease outcomes and T2D) (9).

In the literature, categorization of meat types varied. The most common meat categories were unprocessed red meat, processed red meat, total red meat (including unprocessed and processed red meat), processed meat (including processed red meat and poultry), and poultry. When in an SR, unprocessed red meat and processed red meat were analyzed separately, the possible combined results of total red meat were not considered unless the evidence was substantially stronger for total red meat. Similarly, combined red and processed meat results were reported only if results from more refined categories were unavailable.

The number of cases included in an SR/meta-analysis was reported (in text and in Table 1) for each analysis if it was traceable from the publication. Otherwise, the number of participants was reported instead.

## Diet intake in Nordic and Baltic countries

The average reported meat intake varies between the Nordic and Baltic countries, roughly between 100 and 200 g/day, with significant variation also in the within-country mean intakes (5). Of the total meat intake, red meat accounts for the majority of the intake, with poultry intake being usually several times lower. Poultry intake in the Nordic countries has, however, increased in recent years (6). The mean intakes of any meat in all countries are higher in men than in women. However, the differences in reporting and definition of meat between the countries make comparisons difficult. There are also no data for comparing national average intake of processed meat separately from total red meat.

## Health outcomes relevant for Nordic and Baltic Countries

### Overall CVD and coronary heart disease

One meta-analysis found that lower intakes of both unprocessed red meat and processed meat were associated with modestly lower risk of CVD mortality (unprocessed red meat: 8 studies/389,528 participants; processed meat: 9 studies/478,128 participants) when compared to higher intakes (19) (Table 1). The associations were found only in the studies with low risk of bias. There was evidence for significant heterogeneity but mainly in the studies with high risk of bias (unprocessed red meat: 4 studies/301,788 participants; processed meat: 5 studies/408,839 participants). No associations or evidence of heterogeneity was found with overall CVD incidence risk (unprocessed red meat: 4 studies/65,736 participants; processed meat: 4 studies/69,186 participants). The results were relatively similar in the dose-response analyses for a reduction of 3 servings/wk of unprocessed or processed meat (Table 1).

A meta-analysis by Bechthold et al. (20) found that higher intakes of both total red meat (3 cohorts/6,659 cases) and processed meat intake (5 cohorts/7,038 cases) were associated with higher risk of coronary heart disease (CHD), without evidence of heterogeneity. Although Bechthold et al. (20) found significant non-linearity for the association between total red meat and CHD, that particular analysis was based only on two cohort studies. Processed meat increased the risk of CHD by 27% and of stroke by 17% per each 50 g/day increase in intake (20). Although not with significant non-linearity, the risk for CHD seemed to increase the most on lower intake levels (up to less than 15 g/day).

A meta-analysis in a *de novo* SR (commissioned by the NNR2023 project) found no association between poultry meat intake (unprocessed and processed) and CVD mortality (6 studies/1,158,411 participants) (9). Heterogeneity between the studies was low. There were too few studies for meta-analyses on poultry intake and CVD or CHD incidences. The primary studies showed no association for CVD incidence (1 study) and inverse association (1 study) or no association (1 study) for CHD incidence.

Based on the evidence from several cohort studies with low risk of bias, little evidence for significant heterogeneity, and evidence for biological plausibility (please see section **Mechanisms**), the strength of evidence is regarded as probable that higher unprocessed red meat and processed meat intake are risk factors for CVD mortality, and total red meat, and processed meat are risk factors for CHD (Table 2). Based on the limited number of studies with low risk of bias, the strength of evidence is regarded as limited – no conclusion that high intake of unprocessed red meat or processed meat is a risk factor for overall CVD.

As assessed in the *de novo* SR, substantial effects of poultry meat intake on CVD incidence or mortality were regarded as unlikely.

### Myocardial infarction

One meta-analysis investigated the association between lower intake of unprocessed red meat and processed meat with myocardial infarction and found one prospective cohort study (19). The cohort study (55,171 participants) suggested that lower intakes of both unprocessed red meat and processed meat were associated with modestly lower risk of myocardial infarction.

Based on the limited number of studies, the strength of evidence is regarded as limited – no conclusion that higher unprocessed red meat intake or processed meat intake is a risk factor for myocardial infarction.

### Stroke

In the meta-analysis by Zeraatkar et al. (19), lower intake of unprocessed red meat (any stroke: 6 cohorts/102,024 participants; fatal stroke: 3 cohorts/671,259 participants) and processed meat (any stroke: 6 cohorts/101,861; fatal stroke: 2 studies/571,378 participants) was associated with a modestly lower risk of any stroke and fatal stroke (19). There was little evidence of heterogeneity.

Similar findings were observed in a meta-analysis by Bechthold et al., who found a higher risk of any stroke with higher intake of total red meat (7 cohorts/10,541 cases) or processed meat (6 cohorts/9,492 cases) (20). For the risk of stroke, each 100 g/day increase in total red meat intake increased the risk by 15%. Most studies were considered to have a low risk of bias. There was heterogeneity only for processed meat and only in a dose-response analysis, not in the analysis of extreme categories. In the dose-response analysis, the association with processed meat intake was mainly found in the studies conducted in the USA, with no significant heterogeneity, but not in the European studies.

The *de novo* SR by Ramel et al. found only two primary studies on the association between poultry meat and stroke incidence, which was too few to be combined in meta-analyses (9). One of the primary studies found a lower incidence with higher poultry intake and the other found no association. The risk of bias in the studies was moderate or serious. The same SR found two primary studies (with moderate risk of bias) that did not find association between poultry meat intake and stroke mortality.

Based on the moderate number of studies with low risk of bias, no evidence for unexplained heterogeneity, and with evidence for biologic plausibility, the strength of evidence is regarded as probable that higher unprocessed red meat, total red meat, and processed meat intake are risk factors for any stroke. Based on the limited number of studies with low risk of bias, the strength of evidence is regarded as limited – no conclusion for fatal stroke.

As assessed in the *de novo* SR, the strength of evidence is regarded as limited – no conclusion for poultry meat and stroke incidence and mortality.

### Heart failure

Bechthold et al. observed a higher risk of heart failure with higher intake of total red meat (5 cohorts/9,229 cases) and processed meat (3 cohorts/7,077 cases), although there was evidence for non-linearity (20). No heterogeneity was observed for either of the meat types.

Another meta-analysis also found a higher risk of heart failure with higher intake of processed meat (5 cohorts) but did not find an association with unprocessed red meat intake (5 cohorts) (21). There was evidence of heterogeneity in the analyses with processed meat intake, which was mainly explained by the geographic location. The association with increased risk was stronger in the European studies than in the US studies.

Based on the moderate number of studies with little evidence for unexplained heterogeneity and with evidence for biological plausibility, the strength of evidence is regarded as limited – suggesting that higher intake of total red meat and processed meat is a risk factor for heart failure.

### Hypertension

Meta-analyses of prospective cohort studies found an increased risk of hypertension with higher unprocessed red meat and processed red meat intakes (both meat types: 5 cohorts/23,854 cases (22), total red meat intake: 7 cohorts/97,745 cases (23), and poultry intake: 6 cohorts/14,739 cases (22)). All the associations were with significant unexplained heterogeneity, and the directions of the associations were not always consistent.

Based on the significant unexplained heterogeneity and inconsistent findings, the strength of evidence is regarded as limited – no conclusion that red meat (whether unprocessed or processed), processed meat, or poultry increases the risk of hypertension.

### Type 2 diabetes

A meta-analysis of prospective cohort studies found a reduced risk of T2D with lower intake of unprocessed red meat (12 cohorts/>211,467 participants) and processed meat (19 cohorts/>25,032 participants) (19). Zeraatkar et al. (19) found that the reduction of unprocessed red meat by 3 serving/week (1 serving = 120 g) reduced the risk of T2D by approximately 10%, which equals to approximately 20% reduction in risk by 100 g/day reduction in intake, assuming linear association between the intake and the risk. The association was stronger in low risk of bias studies (*n* = 8) and with no heterogeneity for unprocessed red meat, but the association was weaker for processed red meat with unexplained between-study heterogeneity (19). Another meta-analysis (14 cohorts) also found a higher risk of T2D with higher combined unprocessed and processed red meat (45,702 cases) and processed meat (43,781 cases) intakes, but with significant unexplained between-study heterogeneity (24). Schwingshackl et al. found a 17% increase in risk of T2D for each 100 g/day increase in total red meat intake (24).

A meta-analysis of seven studies (388,283 participants) in the *de novo* SR by Ramel et al. found no association between total poultry meat intake (unprocessed and processed) and risk of T2D (9). There was significant unexplained heterogeneity between the studies. Two of the primary studies investigated unprocessed and processed poultry meat separately (25, 26). The results were inconsistent for both meat types (unprocessed and processed poultry): processed poultry meat was associated with increased risk (1 study) or no risk (1 study), and unprocessed poultry with decreased risk (1 study) or no risk (1 study).

Despite the existing evidence on several low-risk-of-bias cohort studies, dose-response association, and lack of between-study heterogeneity, the lack of effect of red meat on surrogate markers such as blood glucose or insulin concentrations, or a marker of insulin resistance (HOMA-IR) in randomized controlled trials (RCTs) of mainly unprocessed red meat (please see section 6: Mechanisms) led to the strength of evidence regarded as limited – no conclusion that unprocessed red meat increases the risk of T2D. Based on large number of studies with consistent results but significant unexplained heterogeneity, the strength of evidence is regarded as limited – suggesting that processed meat increases the risk of T2D.

As assessed in the *de novo* SR, substantial effects of poultry meat intake on T2D were regarded as unlikely.

### Cancer

#### Colorectal cancer

Both WCRF in their Continuous Update Project and IARC reviewed the available evidence on meat intake and several cancer sites (2, 3, 10). They found that, both, unprocessed red meat and processed meat consumption were associated with increased risk for CRC. The increase in the risk of CRC was 12% for each 100 g/day increase in unprocessed red meat intake (2, 3). The risk for CRC increased by 16% per each 50 g/day increase in the intake of processed meat (2, 3). In the meta-analyses by WCRF, there was no heterogeneity between the studies. IARC concluded based on the large amount of data, strength of association, and consistency across cohort studies in different populations, that there is sufficient evidence in humans that processed meat consumption is a cause of colorectal cancer (10). For unprocessed red meat, IARC concluded that the positive causal interpretation ‘is credible but chance, bias or confounding could not be ruled out’.

As assessed by WCRF and IARC, the strength of evidence is regarded as convincing that processed meat increases the risk of CRC. Based on the conclusions of the IARC and WCRF, the strength of evidence is regarded as probable that unprocessed red meat increases the risk of CRC.

#### Lung cancer

WCRF (2018) found that total red meat intake was associated with increased risk for lung cancer, but there was significant between-study heterogeneity (2, 3). Heterogeneity decreased in analyses by sex, although the number of studies with data available separately for both sexes was small. The association between total red meat and risk of lung cancer persisted in men but not in women. Processed meat was also associated with increased risk of lung cancer with no apparent between-study heterogeneity, but the association was statistically significant in only one of the primary studies. Poultry intake was associated with reduced risk of lung cancer, with no between-study heterogeneity, but only one primary study showed a significant result. The findings of the IARC (2018) regarding red and processed meat were similar to the findings of WCRF (2018), but IARC did not express an evaluation of the strength of evidence (2, 3, 10).

As assessed by WCRF, the strength of evidence is regarded as limited – suggesting that total red meat and processed meat increase the risk of lung cancer and limited – no conclusion evidence that poultry intake decreases the risk of lung cancer.

#### Other cancers

WCRF (2018) found that total red meat was associated with pancreatic cancer and nasopharyngeal cancer (based on a meta-analysis by Li et al. (27)) (2, 3). Large unexplained between-study heterogeneity was present, and the results were based on case-control studies only. WCRF (2018) also found that processed meat was associated with increased risk of pancreatic, nasopharyngeal, esophageal, and stomach cancers (2, 3). No heterogeneity was detected between the studies for any of the cancer types. However, as stated by IARC (2018) on pancreatic, esophageal, and stomach cancers, modest number of studies prevented ruling out chance, bias, and confounding (10).

As assessed by WCRF, strength of evidence is regarded as limited – suggesting that red meat (unprocessed, processed, or both) increases the risk of pancreatic and nasopharyngeal cancers, and that processed meat increases the risk of esophageal, nasopharyngeal, stomach, and pancreatic cancers.

WCRF (2018) did not find sufficient evidence to conclude on the associations between different meat types and cancers of breast, skin, bladder, cervical, gallbladder, kidney, liver, endometrial, ovarian, prostate, mouth, pharynx, and larynx (2, 3). IARC (2018), in addition, did not find sufficient evidence to conclude on the associations between red meat (whether unprocessed or processed) or processed meat intake and cancers of non-Hodgin’s lymphoma, leukemia, and brain (10). No SRs other than those analyzed by WCRF (2018) with adequate quality were found regarding poultry intake and any cancer site. In addition to the cancer sites covered by WCRF and IARC, the literature search returned only an SR on thyroid cancer, which found no association with total meat intake (28).

#### Total cancer incidence and mortality

A meta-analysis of prospective cohort studies found that lower intake of unprocessed red meat (3 cohorts, 875,290 participants) and processed meat (6 cohorts, 1,198,234 participants) was associated with decreased cancer mortality but not with cancer incidence (29). For processed meat, between-study heterogeneity was large but not in studies with low risk of bias. For both, unprocessed red meat and processed meat, the result was significant in only one primary study. One meta-analysis on poultry and total cancer mortality with prospective cohort studies (8 cohorts/257 – 9861 cases in the original studies) found a borderline decreased total cancer mortality in high versus low consumption of poultry with no dose-response association (30).

Based on the limited number of studies with low risk of bias and inconsistent findings, the strength of evidence is regarded as limited – no conclusion that unprocessed red meat and processed meat increase the risk of total cancer mortality. Based on the weak association and lack of dose-response relationship, the strength of evidence is regarded as limited – no conclusion that poultry decreases the risk of total cancer mortality.

### Total mortality

In the meta-analysis by Zeraatkar et al. (19), lower intakes of both unprocessed red meat (9 cohorts/413,760 participants) and processed meat (10 cohorts/>696,822 participants) were associated with a modestly lower risk of all-cause mortality (19). The associations were observed mainly in the studies with low risk of bias. There was evidence of significant heterogeneity between the studies, but the sources of heterogeneity were not studied.

Another meta-analysis by Schwingshackl et al. found that higher total red meat intake (12 cohorts/177,655 cases) and especially intake of processed meat (7 cohorts/143,572 cases) were associated with higher risk of all-cause mortality (31). There was evidence of significant heterogeneity that also persisted in the subgroup analyses.

Based on the large number of studies and with evidence for biologic plausibility, but with significant unexplained heterogeneity, the strength of evidence is regarded as limited – suggesting that higher unprocessed and total red meat and processed meat intake increase the risk of all-cause mortality.

### Other health outcomes

Because of the limited number of studies, no conclusion was possible regarding the association of meat intake with obesity, mental health, metabolic syndrome, chronic obstructive pulmonary disease, gout, chronic kidney disease, gestational diabetes, or anemia. However, the results of the SRs studying the association between meat intake and these outcomes are presented in Table 1 (19, 32–38).

### Note of studies published after the first literature search

After our first draft of the paper, an SR has been published on the association between unprocessed red meat intake and CRC, breast cancer, T2D, ischemic heart disease, ischemic stroke, and hemorrhagic stroke (39). The authors used a novel method for the assessment of uncertainty intervals in meta-analyses and ended up with higher uncertainty in the associations between unprocessed red meat intake and the disease outcomes compared to many previous SRs. The methodology has not, until to date, been commonly accepted by the scientific community (40). Therefore, we did not take the results into account in our conclusions. Another recent meta-analysis (41) generally supports the conclusions of the present scoping review.

## Mechanisms

Red meat is a source of nutrients, such as heme iron, carnitine, and SFA which, in large amounts, may have harmful health effects. Furthermore, processing and cooking of meat have the potential to produce potentially harmful compounds such as N-nitroso compounds (NOCs), heterocyclic amines (HCA), polycyclic aromatic hydrocarbons (PAH), N-glycolylneuraminic acid (Neu5Gc) (42), and advanced glycation end products (AGE) (43). IARC (2018) has classified processed meat as carcinogenic and unprocessed red meat as probably carcinogenic to humans (10). Here, we go through literature regarding suggested mechanisms mediating the potential effects of meat on those chronic diseases, for which there was evidence from observational studies on the association with meat intake (colorectal cancer, cardiovascular outcomes, and T2D).

### Colorectal cancer

Processing and cooking of meat produce potential carcinogens, such as PAH, NOC, and HCA. For example, in Danish studies, concentrations of PAH and HCA increased when barbequing beef, pork, and poultry (44, 45). Different compounds of PAH and NOC were formed during barbecuing depending on the meat type (beef, pork, or poultry). NOCs cause tumors in a variety of animal species and could cause tumors in humans (46). Red meat increases the amount of NOCs in human feces, suggesting also endogenous production, for example, by bacterial activity or by the effect of heme iron of meat (47). Two recent meta-analyses concluded that the consumption of HCA was positively associated with colorectal adenomas (48, 49). Heme iron mediates the formation of lipid peroxidation and NOC in the colon, which can cause DNA damage (42). WCRF has concluded, based on the evaluation of the literature, that there is suggestive evidence on the association between heme iron and CRC (2, 3). Long-term exposure of a compound Neu5Gc, rich in red meat, resulted in an increased incidence of carcinomas in mice (50). SFA in meat products is not a likely explanation for the increased CRC risk because in a recent meta-analysis SFA intake was not associated with CRC (51).

### Blood pressure

The potential effects of red and processed meat could be mediated by salt or heme iron because they both affect the vascular system (52, 53). However, recent SRs and meta-analyses of RCTs have not found an effect of replacing red meat with other food groups on blood pressure (54, 55).

### Glycaemia

Several dietary components of red and processed meat, such as SFA, advanced glycation end products, nitrites and nitrates, heme iron, Trimethylamine-N-oxide (TMAO), branched chain amino acids, or endocrine disruptors, could enhance the development of glycaemia. They can influence glucose and insulin metabolism through affecting adipocyte and muscle cell metabolism, by increasing inflammation and oxidative stress, or through effects on pancreatic β-cell and liver function (56). Meta-analyses of prospective studies suggest an association between serum ferritin and risk of T2D and between heme iron intake and risk of T2D (4, 57, 58). Iron causes oxidative stress, which could inhibit insulin binding (59). Elevated iron concentrations can increase glucose production and output (60) and interfere with hepatic glucose utilization, and glucose metabolism of adipocytes (61) and muscle tissue (62).

However, a meta-analysis of RCTs did not find any effect of red meat on blood glucose concentrations, blood insulin concentrations, HOMA-IR, HbA1c, C-reactive protein (CRP), interleukin 6 (IL-6), or tumor necrosis factor alpha (TNF-alpha) (63). In RCTs, red meat has usually been unprocessed, whereas processed meat intake has been tested less frequently.

### Inflammation

As mentioned earlier, RCTs do not support short-term effects of red meat on inflammation markers (63). Association between red meat and chronic inflammation in observational studies may be confounded by excess body weight (64) or mediated by visceral adiposity resulting from a high SFA diet (65). Other dietary factors, such as fruits, vegetables, and whole grains, may also interact with red meat in the association with inflammation (66).

### Serum lipid profile

A meta-analysis of RCTs found no evidence that red meat would have an adverse impact on blood concentrations of total or high-density lipoprotein cholesterol (HDL, apolipoproteins A1 and B, or triglycerides (54, 67, 68)). For low-density lipoprotein cholesterol (LDL), one meta-analysis of RCTs found no effect (67), whereas another found an adverse effect (68). Guasch-Ferre et al. (54) also found that substitution of red meat by plant foods (soy, nuts, and legumes) had a favorable effect on total and LDL cholesterol concentrations. Both unprocessed and processed red meat often contain large amounts of SFA. The current evidence suggests that reducing SFA, especially when replaced with cis-polyunsaturated fatty acids or cis-monounsaturated fatty acids, improves serum lipid profile and, more specifically, decreases total and LDL cholesterol levels (69). The results of a recent Cochrane SR suggest that reducing SFA for at least two years could reduce the risk of cardiovascular events (70).

### Trimethylamine-N-oxide

Meat contains high amounts of carnitine and choline, which are precursors of TMAO. Red meat intake seems to increase blood concentrations of TMAO (71). High TMAO levels have been associated with increased risk of atherosclerosis and major cardiovascular events (72,73). A recent cross-over RCT found that plant-based alternative meat products decreased TMAO levels compared to animal meat (74). TMAO has also been associated with cancer, potentially through promoting inflammation, oxidative stress, DNA damage, and disruption in protein folding (75). However, it is difficult to interpret whether the effects of meat intake on TMAO have an impact on disease risk, because fish, which contains TMAO, increases circulating TMAO concentration more than red meat (76) but does not increase the risk of CVD. Furthermore, choline is considered an essential nutrient that is required for normal liver and brain function (7).

## Food-based dietary guidelines

There is strong evidence that processed meat intake increases the risk of CRC and probable evidence that unprocessed red meat intake increases the risk of CRC. Several potentially carcinogenic compounds are formed in processing and heating red meat.

There is probable evidence that unprocessed red meat and processed meat intake are risk factors for CVD mortality, and stroke, and that total red meat and processed meat are risk factors for CHD. Based on the current evidence, sodium of processed meat through its effect on blood pressure and SFA of unprocessed and processed red meat through its effect on blood lipids are potential candidates as mediators of the effect.

### Data gaps for future research

One of the main issues is that in most meta-analyses of observational cohort studies, there is little information on food substitution analyses with other protein sources, although this would be very relevant for public health guidelines. In other words, if the intake of meat is reduced, what (protein-containing) foods should be added to the diet? Meta-analyses of observational studies have not commonly addressed this important question, although some original studies have included substitution analyses.

Although cognitive decline shares many of the same risk factors as cardiometabolic diseases, especially processed meat intake has been associated with higher risk of these diseases, currently, there is insufficient data on the impact of meat intake on the risk of cognitive decline. Some observational studies suggest that the consumption of unprocessed meat may have a favorable relationship and processed meat have an unfavorable relationship with cognitive performance (77), but the findings are inconsistent, and a comprehensive synthesis of the longitudinal relationship between the intake of different types of red and white meat and risk of cognitive decline is lacking.

There are also several other outstanding questions, for which comprehensive research data are lacking. These include (in no particular order) the following: Is red meat from game or grass-fed animals healthier than the red meat from animals raised by conventional intensive agriculture? What is the health impact of organ meat intake? What is the health impact of the different cooking methods of meat? What is the impact of lean versus fatty meat on the risk of diseases? Do the health impacts of red meat from different species (e.g. beef, pork, and sheep) differ from each other?

### Limitations

One of the main limitations is that, as with most dietary factors, there are no long-term RCTs that would have investigated the effects of consuming different kinds of meat on disease outcomes. Such studies would provide the highest quality of evidence but will likely never be conducted due to financial, practical, and logistic reasons. Therefore, the evidence is based on short-term RCT with disease risk factors or on observational studies. The interpretation of findings from RCTs can be challenging because the results may depend on the comparison food that replaces meat in the diet. Most RCTs last only a few weeks or months, which may be too short a time to observe significant effects on disease risk factors. It is possible that, for example, high blood pressure or glycaemia develops over a period of as long as years or decades. Even in short-term RCTs, the attrition may be high, especially if the participants are required to make large changes to their typical diets. In RCTs, the meat is also often minimally processed lean meat, and therefore, the evidence of processed meat on, for example, blood pressure and glycaemia is limited (55, 63). On the other hand, observational studies do not provide evidence for causality and may be biased due to residual confounding, reverse causation, and difficulty in estimating dietary intakes accurately and repeatedly. The significant heterogeneity in many meta-analyses of observational studies of meat intake and risk of diseases may partly be explained by these issues. A limitation is also that very few cohort studies collect information on long-term diet.

Another limitation is that the definition of red meat is not always the same. In some studies, red meat refers to only unprocessed red meat, whereas in other studies, red meat refers to a mixture of unprocessed and processed red meat. This may be one cause of the heterogeneity often observed in meta-analyses. There is also little evidence for the associations between meat from different animal species and health-related outcomes. Many studies have combined intakes of processed red and processed white meat when they have analyzed the relationships between processed meat intake and risk of disease. Therefore, there are not sufficient data to conclude whether processed white meat intake is as harmful as intake of processed red meat.

The quality of the majority of the SRs on the association between meat and health outcomes was rated as critically low (according to AMSTAR 2 evaluation). The main limitations that led to the rating ‘critically low’ were failure to preregister the plan for the SR and restricting the literature search to articles written in English. Addressing these issues in future SRs would improve the quality with relatively little additional effort from the authors.

## Supplementary Material


