# Supplementary material for: Meat and meat products – a scoping review for Nordic Nutrition Recommendations 2023
Source: Food Nutr Res. 2024 Feb 21;68:10.29219/fnr.v68.10538. doi: 10.29219/fnr.v68.10538 (PMC10916397; doi:10.29219/fnr.v68.10538)
Supplement: Supplementary file 1 [file FNR-68-10538-s001.docx]

Supplementary table 1. List of excluded studies in Meat and meat products – a scoping review for Nordic Nutrition Recommendations 2023.

| **1st author (year)** | **Exposure** | **Outcome(s)** | **Main findings, if meta-analysis conducted** | **Reason for not including in the chapter** |
| --- | --- | --- | --- | --- |
| Feskens (2013) | Total meat, red meat, processed meat, poultry | T1D, T2D, CHD, stroke | Higher risk of T2D and CHD with higher intake of unprocessed red meat and especially of processed meat. No association with poultry. Meta-analysis not done for T1D because only few studies available. | More recent or more comprehensive meta-analyses available |
| Fardet (2014) | Red and processed meat, poultry | Major diet-related chronic diseases | Red/processed meats associated with increased risk of diet related chronic diseases. | Umbrella review |
| Evans (2019) | Meat | Gout | No results reported on meat – gout association. | No results reported on meat – gout association. |
| Singh (2011) | Meat | Gout | Meat intake increased the risk of incident gout. | More recent or more comprehensive meta-analyses available |
| Kim (2018) | Total meat, red meat, processed meat, poultry | Metabolic syndrome | Total meat intake and especially intakes of red meat and processed meat were positively associated with risk of metabolic syndrome. White meat intake was inversely associated with the risk. | More recent or more comprehensive meta-analyses available |
| Chalvon-Demersay (2017) | Animal protein sources | Metabolic syndrome / obesity | No conclusion on meat – metabolic syndrome association or meat – obesity association. | More recent or more comprehensive meta-analyses available |
| Jakobsen (2021) | Unprocessed and processed meat | CVD, CHD, stroke | The meta-evidence was graded moderate for a positive association between unprocessed red meat and stroke and moderate for a positive association between processed meat and CHD and stroke. | Umbrella review |
| Papp (2021) | Poultry | CVD, CHD, stroke, all-cause mortality | Higher poultry intake associated with modestly lower risk of all-cause mortality. Substituting red and/or processed meat with poultry was inversely associated with all investigated outcomes. | *de* novo NNR2022 systematic review available |
| Lupoli (2021) | White meat | All-cause mortality, CVD | White meat consumption had an inverse association with all-cause mortality and neutral association with CVD. | *de* novo NNR2022 systematic review available |
| Papier (2021) | Unprocessed red meat, processed meat, poultry | Ischemic heart disease | Higher intake of unprocessed red meat and processed meat were associated with a higher risk of ischemic heart disease. Poultry was not associated. | Inappropriate use of fixed-effect modeling in meta-analysis. |
| Händel (2019) | Processed meat | Chronic disease morbidity and mortality | The scientific  quality of the systematic reviews reporting positive associations between processed meat  intake and risk of various cancers, T2D and CVD is moderate, and the results from case-control studies suggest more often a positive association than the results from cohort studies. The overall certainty in the evidence was very low across all individual outcomes, due to  serious risk of bias and imprecision. | Umbrella review |
| Zeraatkar (2019) | Red meat | All-cause mortality, cardiovascular mortality, adverse cardiometabolic events and major morbidity, cancer mortality and incidence, quality of life, and surrogate outcomes | Low- to very-low-certainty evidence suggests that diets restricted in red meat may have little or no effect on major cardiometabolic outcomes and cancer mortality and incidence. | SR of RCTs, which is based mainly on a trial aiming for low-fat dietary pattern where red meat intake had a minimal role. (The part with anemia as an outcomes was included in the chapter). |
| Vernooij (2019) | Dietary patterns with high vs. low red and processed meat | Cardiometabolic outcomes, cancer outcomes | Dietary patterns with less red and processed meat associated with modestly lower risk of cardiometabolic and cancer outcomes | Investigated dietary patterns |
| Kwok (2019) | Red meat, processed meat, white meat | CVD, all-cause mortality | All-cause mortality: processed meat appeared to be harmful. Cardiovascular disease: red meat and processed meat were associated with harmful effects. | Umbrella review |
| Mohammadi (2018) | Poultry | Stroke | Poultry intake not associated with stroke risk | *de* novo NNR2022 systematic review available |
| Deng (2018) | Red and processed meat | Stroke | A high consumption of red and/or processed meat was associated with increased stroke risk | Umbrella review |
| Yip (2018) | Fresh and/or processed poultry and/or red meat | Several health outcomes | Processed meat intake was associated with higher risk for esophageal cancer, stomach cancer, CHD, diabetes, colon cancer incidences, and 1 CVD mortality. Total red meat intake was associated with higher risk for endometrial, esophageal, and lung cancer incidences. In addition, 14 statistically significant associations in terms of high vs low meat intake relative risks were also identified. Total red meat intakes were found negatively associated with CVD and cancer mortalities, and poultry meat intakes were found negatively associated with all-cause and cancer mortalities, and rectal cancer incidences in low meat consumption Asian countries. | Umbrella review |
| Iacoviello (2018) | Red and processed meat | Stroke | Strong evidence that the consumption of red and processed meat is associated with an increased risk of ischemic stroke. | No meta-analysis. SR with meta-analysis available |
| Nowson (2018) | Red and processed meat | Non-fatal cardiovascular events, cognition, mental health, falls and fractures, physical health (muscle mass, strength) and frailty | No conclusions on meat. | No associations between meat intake and ICD classified outcomes reported |
| Kim (2017) | Total meat, red meat, processed meat, white meat | Stroke | Intakes of total meat, red meat and processed meat associated with higher risk of total stroke, poultry intake with lower risk. No associations with stroke mortality. | More recent or more comprehensive meta-analyses available |
| Wolk (2017) | Red meat | Diabetes, coronary heart disease, heart failure, stroke and cancer at several sites, and mortality | High consumption of red meat, and especially processed meat, is associated with an increased risk of several major chronic diseases and preterm mortality. | Umbrella review |
| Yang (2016) | Red and processed meat | Stroke | Total red meat intake was associated with higher risk of total stroke, cerebral infarction and ischemic stroke. Processed red meat intake was associated with higher risk of total stroke and intake of fresh red meat with higher risk of total and ischemic stroke. No type of meat was associated with hemorrhagic stroke. | More recent or more comprehensive meta-analyses available |
| Lippi (2015) | Red meat | Ischemic heart disease | Larger intake of red meat was found to be a risk factor for IHD in four studies, no association was found in five other studies. | No meta-analysis. SR with meta-analysis available |
| Abete (2014) | Total meat, red meat, processed meat, white meat | All-cause, CVD and ischemic heart disease mortality | Processed meat intake was associated with higher risk of all-cause and CVD mortality and red meat consumption with higher CVD mortality risk. Total and white meat intakes were not associated with the risk and not any type of meat was associated with ischemic heart disease mortality. | More recent or more comprehensive meta-analyses available |
| Chen (2013) | Red and processed meat | Stroke | Intakes of total meat, red meat and processed meat were associated with a higher risk of total and ischemic stroke, but not with hemorrhagic stroke. | More recent or more comprehensive meta-analyses available |
| Åkesson (2013) | Red and processed meat | CVD, T2D, inflammatory factors, colorectal, prostate, breast cancers, bone health, iron status | No conclusion on red and processed meat intake in relation to cardiovascular disease (CVD) and iron status. The WCRF/AICR concludes that red and processed meat is a convincing cause of colorectal cancer. | No meta-analysis. SR with meta-analysis available |
| Kaluza (2012) | Total meat, red meat, processed meat | Stroke | Intakes of total meat, fresh meat and processed meat were associated with a higher risk of total stroke and ischemic stroke, but not with hemorrhagic stroke | More recent or more comprehensive meta-analyses available |
| Yang (2020) | Total meat, red meat, processed meat, poultry | T2D | Intakes of total meat, red meat and processed meat were associated with a higher risk, poultry intake had no association | More recent or more comprehensive meta-analyses available |
| Fan (2019) | Red meat, processed meat, poultry | T2D | Intakes of red meat and processed meat were associated with a higher risk. Also poultry intake was very modestly associated with a higher risk | More recent or more comprehensive meta-analyses available |
| Micha (2012) | Unprocessed red meat and processed red meat | CHD, T2D | Processed meat intake was associated with a higher risk of CHD and T2D, unprocessed red meat intake with higher risk of T2D | More recent or more comprehensive meta-analyses available |
| Dobersek (2021) | Meat avoidance | Depression or depressive symptoms/disorders, Anxiety or anxiety-related symptoms/disorders, Deliberate self-harm (e.g., attempted suicide), Mood/stress perception, Affective well-being/Quality of life (QoL) | Meat-abstention was associated with poorer psychological health in majority but not all of the studies. | Mainly cross-sectional studies. |
| Nucci (2020) | Red and processed meat (combined) | Depression | Higher meat intake was associated with a higher risk of depression | Unclear statistical analyses |
| Shareghfarid (2020) | Red and processed meat | ADHD | Processed meat consumption was associated with more complicated symptoms in children. | Systematic review with only one cross-sectional study on meat - ADHD association. |
| Zhang (2020) | Meat | Cognitive function | Higher meat intake was associated with a lower odds for cognitive disorders, but with evidence for publication bias | No ICD classified outcomes |
| An (2019) | Beef | Cognition |  | No ICD classified outcomes |
| Solfrizzi (2017) | Meat (and other dietary factors) | Alzheimer’s disease |  | No studies on meat and Alzheimer’s disease |
| Cohen (2016) | Red and processed meat | Cognitive function | Red/processed meats were inversely associated with executive functioning. | No ICD classified outcomes |
| Bennett (2015) | Red and processed meat (and other food groups and lifestyle factors) | Small intestine adenocarcinoma | High intakes of red/processed meat were associated with increased small intestine adenocarcinoma risk. | No meta-analysis for dietary factors. SR with meta-analysis available |
| Zhang (2017) | Red and processed meat | Renal carcinoma | Positive association between red and processed meat intake and renal carcinoma risk | Qualified systematic review from the World Cancer Research Fund available |
| Crane (2014) | Total meat, red meat, processed meat, poultry | Ovarian cancer | No association. | Qualified systematic review from the World Cancer Research Fund available |
| Wallin (2011) | Red and processed meat | Ovarian cancer | Red and processed meat consumption was not associated with risk of ovarian cancer. | Qualified systematic review from the World Cancer Research Fund available |
| George (2021) | Red meat, Processed meat, white meat | Hepatocellular carcinoma | Intake of poultry was associated with a reduced risk of HCC. | Qualified systematic review from the World Cancer Research Fund available |
| Luo (2014) | Total meat, red meat, processed meat, white meat | Hepatocellular carcinoma | High level of white meat or fish consumption were associated with reduced risk of HCC, while intake of red meat, processed meat or total meat were not associated with HCC risk. | Qualified systematic review from the World Cancer Research Fund available |
| Xu (2014) | Total meat, red meat, white meat | Oral cavity and oropharynx cancer | High consumption of processed meat was associated with an increased risk of oral cavity and oropharynx cancer, while there was no significantly association between total meat, red meat or white meat and the risk of oral cavity and oropharynx cancer. | Qualified systematic review from the World Cancer Research Fund available |
| Gathirua-Mwangi (2014) | Meat | Prostate cancer | An inconsistent association was observed for intake of total meat. | Qualified systematic review from the World Cancer Research Fund available |
| Bylsma (2015) | Red meat, processed meat | Prostate cancer | Processed meat was associated with increased risk while total red meat, and fresh red meat consumption were not. | Qualified systematic review from the World Cancer Research Fund available |
| Mandair (2016) | Red meat, processed meat, poultry | Prostate cancer | Red meat, especially well-done red meat, associated with increased risk of prostate cancer. | Qualified systematic review from the World Cancer Research Fund available |
| Shin (2019) | Animal-based foods | Prostate cancer | No clear association between meat intake and increased prostate cancer risk. | Qualified systematic review from the World Cancer Research Fund available |
| Fallahzadeh (2014) | Red meat, processed red meat | non-Hodgkin lymphoma | Higher intake of red meat and processed red meat were associated with a higher risk of non-Hodgkin lymphoma | More recent or more comprehensive meta-analysis available |
| Yang (2015) | Red meat, processed meat | non-Hodgkin lymphoma | Higher intake of red meat and processed red meat were associated with a higher risk of non-Hodgkin lymphoma | More recent or more comprehensive meta-analysis available |
| Caini (2016) | Red meat, processed meat, white meat | non-Hodgkin lymphoma, multiple myeloma | Higher red meat intake was associated with a higher risk of non-Hodgkin lymphoma | More recent or more comprehensive meta-analysis available |
| Wang (2012) | Total meat, red meat, processed meat | Bladder cancer | Overall meat intake not related to the risk of bladder cancer while high red and processed meat intake were associated with increased risk. | Qualified systematic review from the World Cancer Research Fund available |
| Crippa (2018) | Red meat, processed meat | Bladder cancer | Red meat intake was associated with higher bladder cancer risk in case-control studies but not in cohort studies. Processed meat intake was associated with bladder cancer also separately in case-control and cohort studies. | Qualified systematic review from the World Cancer Research Fund available |
| Paluszkiewicz (2012) | Red meat, processed meat, poultry | Pancreatic cancer | Red meat intake was associated with elevated risk of pancreatic cancer in case-control but not in cohort studies. Poultry intake was not associated with pancreatic cancer risk. | Qualified systematic review from the World Cancer Research Fund available |
| Larsson (2012) | Red meat, processed meat | Pancreatic cancer | Processed meat consumption was positively associated with pancreatic cancer risk. Red meat consumption was associated with an increased risk of pancreatic cancer in men. | Qualified systematic review from the World Cancer Research Fund available |
| Zhao (2017a) | Red meat, processed meat | Pancreatic cancer | Case-control but not cohort studies associated consumption of red and processed meat with risk of pancreatic cancer. | Qualified systematic review from the World Cancer Research Fund available |
| Gao (2020) | Poultry | Pancreatic cancer | Poultry intake was associated with higher risk of pancreatic cancer in cohort studies. | Qualified systematic review from the World Cancer Research Fund available |
| Salem (2018) | Red and processed meat | Pancreatic cancer | A positive association for intake of red and processed meat and pancreatic cancer, in men in particular. | Qualified systematic review from the World Cancer Research Fund available |
| Yang (2012) | Total meat, red meat, processed meat, poultry | Lung cancer | A high intake of red meat was associated with increased risk of lung cancer while a high intake of poultry was associated with decreased risk. | Qualified systematic review from the World Cancer Research Fund available |
| Xue (2014) | Red meat, processed meat | Lung cancer | Both red and processed meat consumption showed a positive association with lung cancer risk. | Qualified systematic review from the World Cancer Research Fund available |
| Koutsokera (2013) | Red meat, processed meat | Lung cancer | Evidence from case-control studies suggests a positive association between meat intake and risk of lung cancer, although several more recent studies have presented doubts about these findings. | Qualified systematic review from the World Cancer Research Fund available |
| Gnagnarella (2018) | Red meat, processed meat, white meat, offal | Lung cancer | High consumption of red meat was associated with higher risk of lung cancer. No significant associations between high consumption  of other types of meat, or heterocyclic amines and lung cancer risk were detected. | Qualified systematic review from the World Cancer Research Fund available |
| Guo (2015) | Red meat, processed meat | Breast cancer | Increased intake of red and processed meat associated with an increased risk of breast cancer. | Qualified systematic review from the World Cancer Research Fund available |
| Wu (2016) | Total red meat, fresh red meat, processed meat, poultry | Breast cancer | Red and processed meat were associated with high breast cancer risk whereas poultry was not. | Qualified systematic review from the World Cancer Research Fund available |
| Mourouti (2015) | Meat | Breast cancer | Studies on meat consumption and breast cancer provide inconsistent results. | Qualified systematic review from the World Cancer Research Fund available |
| Anderson (2018) | Red meat, processed meat | Breast cancer | Processed meat consumption was associated with overall and post-menopausal, but not pre-menopausal, breast cancer. Red meat consumption was not associated with breast cancer. | Qualified systematic review from the World Cancer Research Fund available |
| Farvid (2018) | Red meat, processed meat | Breast cancer | High processed meat consumption was associated with increased breast cancer risk. | Qualified systematic review from the World Cancer Research Fund available |
| Kazemi (2021) | Red meat, processed meat, poultry | Breast cancer | Low intakes of red and processed meat were associated with lower risks of breast cancer. | Qualified systematic review from the World Cancer Research Fund available |
| Rezaianzadeh (2018) | Red meat | Breast cancer | Association between red meat consumption and breast  cancer risk. | Qualified systematic review from the World Cancer Research Fund available |
| Saneei (2015) | Total red meat, unprocessed red meat, processed meat | Glioma | Higher unprocessed red meat intake was associated with a higher risk of glioma. No association with processed red meat or total meat intakes. | More recent or more comprehensive meta-analysis available |
| Wei (2015) | Red meat, processed meat | Glioma | Higher processed meat intake was associated with a higher risk of glioma. No association with red meat intake. | More recent or more comprehensive meta-analysis available |
| Zumel-Marne (2019) | Total meat, bacon, non-cured meat, hot dogs | Brain tumors | Higher meat intake during pregnancy associated with higher risk of brain tumors in the offspring. No meta-analyses on other exposures. | Unclear statistical analyses |
| Quach (2017) | Cured meat | Brain tumors | Cured meat intake (by means of exposure to N-nitroso compounds) is associated with both childhood brain tumours and adult onset glioma. | Umbrella review |
| Fang (2015) | Red meat, processed meat, ham, bacon, sausage | Gastric cancer | Elevated consumption of processed meat was associated with increased risk of GC. | Qualified systematic review from the World Cancer Research Fund available |
| Song (2014) | Red meat | Stomach cancer | Positive association but when stratified by design, only in case-control studies. | Qualified systematic review from the World Cancer Research Fund available |
| Zhu (2013) | Red meat, processed meat | Gastric cancer | Consumption of red and/or processed meat were associated with increased gastric cancer risk. For red meat the association was found only in case-control studies. | Qualified systematic review from the World Cancer Research Fund available |
| Zhao (2017b) | Red meat, processed meat | Gastric cancer | Null results on cohort studies but positive associations in case-control studies. | Qualified systematic review from the World Cancer Research Fund available |
| Li (2012) | Red meat, processed meat | Gastric cancer | Processed meat was associated with increased GC risk. | Qualified systematic review from the World Cancer Research Fund available |
| Kim (2019) | Red meat, processed meat, white meat | Gastric cancer | The increase of white meat consumption was associated with reduced risk of gastric cancer, while red or processed meat were associated with increased risk. | Qualified systematic review from the World Cancer Research Fund available |
| Vahid (2021) | Red meat | Gastric cancer | Red meat was associated with increased risk of developing GC. | Qualified systematic review from the World Cancer Research Fund available |
| Huang (2013) | Red meat, processed meat | Esophageal cancer | Consumption of red meat and processed meat were associated with a greater risk of esophageal cancer | Qualified systematic review from the World Cancer Research Fund available |
| Choi (2013) | Red meat, processed meat | Esophageal cancer | Consumption of red meat was associated with a greater risk of esophageal cancer but processed meat only in case-control studies. | Qualified systematic review from the World Cancer Research Fund available |
| Salehi (2013) | Total meat, red meat, processed meat, poultry | Esophageal cancer | Red and processed meat but not total meat or poultry were associated with higher esophageal cancer risk. | Qualified systematic review from the World Cancer Research Fund available |
| Jiang (2016) | Poultry | Esophageal cancer | No association. | Qualified systematic review from the World Cancer Research Fund available |
| Qu (2013) | Red meat, processed meat | Esophageal cancer | Red and processed meat were associated with esophageal cancer risk. | Qualified systematic review from the World Cancer Research Fund available |
| Zhu (2014) | Total meat, red meat, processed meat, poultry | Esophageal cancer | High intake of red meat and low intake of poultry were associated with an increased risk of esophageal squamous cell carcinoma. High meat intake, especially processed meat, was likely to increase esophageal adenocarcinoma risk. | Qualified systematic review from the World Cancer Research Fund available |
| Castro (2017) | Red meat, processed meat, white meat | Esophageal cancer | ESCC risk significantly increased with red and processed meat consumption and decreased with white meat consumption. | Qualified systematic review from the World Cancer Research Fund available |
| Zhao (2020) | Red meat, processed meat | Esophageal cancer | Case-control but not cohort studies associated consumption of red and processed meat with the risk of esophageal cancer. | Qualified systematic review from the World Cancer Research Fund available |
| Lippi (2016) | Red meat, processed meat, poultry | Cancer | Increased risk of cancer in subjects consuming large amounts of red and processed meat, but not in those with high intake of white meat or poultry. | Umbrella review |
| Latino-Martel (2016) | Red meat, processed meat | Cancer | Red meat and processed meat are associated with increased risk of cancer. | Umbrella review |
| Johnson (2017) | Red meat, processed meat, poultry | Cancer | Epidemiology indicates that processed meat products are associated with increased risk of colorectal cancer. Evidence for red meat and for other cancers remains tentative. | Umbrella review |
| Zhao (2016) | Poultry | Colorectal cancer incidence and mortality | Increase in  poultry intake was associated with decreased  incidence of CRC. No association between poultry intake and  CRC mortality. | Qualified systematic review from the World Cancer Research Fund available |
| Ekmekcioglu (2018) | Red meat, processed meat | T2D, CVD, colorectal and other cancers, and all-cause mortality | High intake of red and especially processed meat is associated with higher risk for type 2 diabetes, CRC, total mortality, and CVDs. | Umbrella review |
| Carr (2016) | Beef, pork, lamb, veal, poultry | Colorectal cancer | Beef consumption was associated with an increased risk of CRC and colon cancer, but not with rectal cancer. Higher consumption of lamb, but not pork, was associated with increased risk of CRC. No association was observed for poultry consumption and risk of colorectal adenomas or cancer. | Qualified systematic review from the World Cancer Research Fund available |
| Alexander (2015) | Red meat | Colorectal cancer | A weak elevated summary relative risk was observed. | Qualified systematic review from the World Cancer Research Fund available |
| Shi (2015) | Poultry | Colorectal cancer incidence and mortality | Poultry intake may be moderately associated with reduced incidence of CRC. | Qualified systematic review from the World Cancer Research Fund available |
| van Meer (2013) | Meat | Colorectal cancer mortality | An association between meat intake and increased CRC mortality was found in two ecologic studies; however, two prospective cohort studies did not confirm this association. | Qualified systematic review from the World Cancer Research Fund available |
| Aune (2013) | Red meat, processed meat | Colorectal adenomas | Elevated risk of colorectal adenomas with intake of red and processed meat. | Qualified systematic review from the World Cancer Research Fund available |
| Chan (2011) | Red meat, processed meat | Colorectal cancer | High intake of red and processed meat is associated with increased risk of colorectal, colon and rectal cancers. | Qualified systematic review from the World Cancer Research Fund available |
| Johnson (2013) | Red meat | Colorectal cancer | Increased red meat intake, was associated with moderately increased risk of CRC. | Qualified systematic review from the World Cancer Research Fund available |
| Xu (2013) | Poultry | Colorectal adenomas | Intake of white meat (fish/poultry) is not associated with the risk of CRA. | Qualified systematic review from the World Cancer Research Fund available |
| Zhao (2017c) | Red meat, processed meat | Colorectal cancer | Consumption of red and processed meat was associated with the risk of overall colorectal cancer but not rectal cancer. No associations between red meat consumption and distal colon cancer risk and between processed meat consumption and proximal colon cancer risk. | Qualified systematic review from the World Cancer Research Fund available |
| Alexander (2011) | Red meat | Colorectal cancer | Red meat was associated with CRC with significant heterogeneity and associations were modified by tumor site and sex. | Qualified systematic review from the World Cancer Research Fund available |
| Fardet (2017) | Total meat, red meat, processed meat, poultry | Colorectal cancer | Red/processed meats tend to increase the risk of CRC. | Qualified systematic review from the World Cancer Research Fund available |
| Vargas (2012) | Red meat, processed meat | Colorectal cancer | Based on our current knowledge and level of confidence in the evidence, diet and nutrient recommendations for patients that can be made with confidence for lowering the risk of colorectal cancer include moderating intake of red and processed meats and cooking meats at a safe but lower temperature to avoid charring or blackening the meat. | Qualified systematic review from the World Cancer Research Fund available |
| Vieira (2017) | Red meat, processed meat, poultry | Colorectal cancer | Increase of red and processed meat intake was associated with increased risk of CRC. Intake of poultry was not associated with colorectal cancer risk. | Qualified systematic review from the World Cancer Research Fund available |
| Veettil (2021) | Red meat, processed meat | Colorectal cancer | There was convincing evidence of an association of intake of red meat with the incidence of CRC. | Qualified systematic review from the World Cancer Research Fund available |
| Händel (2020) | Processed meat | Colorectal cancer, colon, and rectal cancer | Processed meat consumption was associated with risk of CRC and colon cancer but not with rectal cancer. | Qualified systematic review from the World Cancer Research Fund available |
| An (2020) | Pork | Body weight / obesity | Among the experimental studies pork intake was associated with a reduction in body weight. Among the observational studies, pork intake was not associated with overweight/obesity. | Included only cross-sectional studies. |
| Grosso (2017) | Total meat, red meat, processed meat | BMI / obesity | Intake of red meat was positively associated with obesity. | Analysis of unadjusted results from cross-sectional studies. |
| Rouhani (2014) | Red and processed meat | Obesity | Red and processed meat intake directly associated with risk of obesity. | Included only cross-sectional studies. |
| Fogelholm (2012) | Total meat, red meat, processed meat, poultry | Weight change |  | No meat - obesity association studied. |
| Granic (2020) | Red meat, processed meat, poultry | Sarcopenia |  | No meat - sarcopenia association studied. |
| Hoorsan (2017) | Chicken, red meat, bacon | Endometriosis | No associations | Unclear statistical analyses (comparison groups not known) |
| He (2020) | Red meat | Non-alcoholic fatty liver disease | Individuals who consumed more red meat may have a significantly increased likelihood of NAFLD. | Unclear statistical analyses (comparison groups not known) and includes mainly cross-sectional studies. |
| Hou (2011) | Meat (and other dietary factors) | Inflammatory bowel disease | High dietary intake of meat was associated with an increased risk of Crohn’s disease and ulcerative colitis. | More recent SR with meta-analysis available. |
| Chapelle (2020) | Meat (and other dietary factors) | Colon or colorectal cancer | An increased incidence of CRC was observed with frequent meat consumption. | Umbrella SR |
| Eleftheriou (2018) | Meat (Mediterranean diet and its components) | All-cause mortality | A positive association was apparent for meat. | Meat types not analyzed separately. A SR with separately analysed meat types available. |
| Wang (2016) | Red and processed meat | Mortality | Higher consumption of total red meat and processed meat associated with an increased risk of total, cardiovascular and cancer mortality. | More recent and more comprehensive meta-analysis available. |
| Larsson (2014) | Red and processed meat | All-cause mortality | High consumption of red meat, especially processed meat, may increase all-cause mortality. | More recent and more comprehensive meta-analysis available. |
| O´Sullivan (2013) | Meat and processed meat | Mortality | High intakes of meat and processed meat were associated with an increased risk of mortality but with a decreased risk in a subanalysis of Asian studies. | More recent and more comprehensive meta-analysis available. |
| Kouvari (2016) | Red and processed meat | Diabetes mellitus | All of studies highlighted the aggravating role of processed meat-products in diabetes incidence, while fresh meat reached significance in only half of them. | Umbrella review |
| Neuenschwander (2019) | Red Meat, well cooked and Processed Meat | Type 2 diabetes | Quality of evidence was high for the association for increased incidence of type 2 diabetes with higher intake of red, processed meat, and bacon. | Umbrella review |
| Toi (2020) | Red and processed meat (and other food grups) | Type 2 diabetes | Unhealthy diets such as high consumption of high glycemic index and glycemic load diet, red meat and processed meat, sugar and artificial sweetened beverages can accelerate the development of T2DM. | Umbrella review |

SR, systematic review. RCT, randomized controlled trial.

**References:**

Abete I, Romaguera D, Vieira AR, Lopez de Munain A, Norat T. Association between total, processed, red and white meat consumption and all-cause, CVD and IHD mortality: a meta-analysis of cohort studies. Br J Nutr. 2014 Sep 14;112(5):762-75.

Alexander DD, Weed DL, Cushing CA, Lowe KA. Meta-analysis of prospective studies of red meat consumption and colorectal cancer. Eur J Cancer Prev. 2011 Jul;20(4):293-307.

Alexander DD, Weed DL, Miller PE, Mohamed MA. Red Meat and Colorectal Cancer: A Quantitative Update on the State of the Epidemiologic Science. J Am Coll Nutr. 2015;34(6):521-43.

An R, Liu J, Liu R. Pork Consumption in Relation to Body Weight and Composition: A Systematic Review and Meta-analysis. Am J Health Behav. 2020 Jul 1;44(4):513-525.

An R, Nickols-Richardson SM, Khan N, Liu J, Liu R, Clarke C. Impact of Beef and Beef Product Intake on Cognition in Children and Young Adults: A Systematic Review. Nutrients. 2019 Aug 3;11(8):1797.

Anderson JJ, Darwis NDM, Mackay DF, Celis-Morales CA, Lyall DM, Sattar N, Gill JMR, Pell JP. Red and processed meat consumption and breast cancer: UK Biobank cohort study and meta-analysis. Eur J Cancer. 2018 Feb;90:73-82.

Aune D, Chan DS, Vieira AR, Navarro Rosenblatt DA, Vieira R, Greenwood DC, Kampman E, Norat T. Red and processed meat intake and risk of colorectal adenomas: a systematic review and meta-analysis of epidemiological studies. Cancer Causes Control. 2013 Apr;24(4):611-27.

Bennett CM, Coleman HG, Veal PG, Cantwell MM, Lau CC, Murray LJ. Lifestyle factors and small intestine adenocarcinoma risk: A systematic review and meta- analysis. Cancer Epidemiol. 2015 Jun;39(3):265-73.

Bylsma LC, Alexander DD. A review and meta-analysis of prospective studies of red and processed meat, meat cooking methods, heme iron, heterocyclic amines and prostate cancer. Nutr J. 2015 Dec 21;14:125.

Caini S, Masala G, Gnagnarella P, Ermini I, Russell-Edu W, Palli D, Gandini S. Food of animal origin and risk of non-Hodgkin lymphoma and multiple myeloma: A review of the literature and meta-analysis. Crit Rev Oncol Hematol. 2016 Apr;100:16-24.

Carr PR, Walter V, Brenner H, Hoffmeister M. Meat subtypes and their association with colorectal cancer: Systematic review and meta-analysis. Int J Cancer. 2016 Jan 15;138(2):293-302.

Castro C, Peleteiro B, Lunet N. Modifiable factors and esophageal cancer: a systematic review of published meta-analyses. J Gastroenterol. 2018 Jan;53(1):37-51.

Chalvon-Demersay T, Azzout-Marniche D, Arfsten J, Egli L, Gaudichon C, Karagounis LG, Tomé D. A Systematic Review of the Effects of Plant Compared with Animal Protein Sources on Features of Metabolic Syndrome. J Nutr. 2017 Mar;147(3):281-292.

Chan DS, Lau R, Aune D, Vieira R, Greenwood DC, Kampman E, Norat T. Red and processed meat and colorectal cancer incidence: meta-analysis of prospective studies. PLoS One. 2011;6(6):e20456.

Chapelle N, Martel M, Toes-Zoutendijk E, Barkun AN, Bardou M. Recent advances in clinical practice: colorectal cancer chemoprevention in the average- risk population. Gut. 2020 Dec;69(12):2244-2255.

Chen GC, Lv DB, Pang Z, Liu QF. Red and processed meat consumption and risk of stroke: a meta-analysis of prospective cohort studies. Eur J Clin Nutr. 2013 Jan;67(1):91-5.

Choi Y, Song S, Song Y, Lee JE. Consumption of red and processed meat and esophageal cancer risk: meta-analysis. World J Gastroenterol. 2013 Feb 21;19(7):1020-9.

Cohen JF, Gorski MT, Gruber SA, Kurdziel LB, Rimm EB. The effect of healthy dietary consumption on executive cognitive functioning in children and adolescents: a systematic review. Br J Nutr. 2016 Sep;116(6):989-1000.

Crane TE, Khulpateea BR, Alberts DS, Basen-Engquist K, Thomson CA. Dietary intake and ovarian cancer risk: a systematic review. Cancer Epidemiol Biomarkers Prev. 2014 Feb;23(2):255-73.

Crippa A, Larsson SC, Discacciati A, Wolk A, Orsini N. Red and processed meat consumption and risk of bladder cancer: a dose-response meta-analysis of epidemiological studies. Eur J Nutr. 2018 Mar;57(2):689-701.

Deng C, Lu Q, Gong B, Li L, Chang L, Fu L, Zhao Y. Stroke and food groups: an overview of systematic reviews and meta-analyses. Public Health Nutr. 2018 Mar;21(4):766-776.

Dobersek U, Wy G, Adkins J, Altmeyer S, Krout K, Lavie CJ, Archer E. Meat and mental health: a systematic review of meat abstention and depression, anxiety, and related phenomena. Crit Rev Food Sci Nutr. 2021;61(4):622-635.

Ekmekcioglu, C., et al. (2018). "Red meat, diseases, and healthy alternatives: A critical review." Critical Reviews in Food Science and Nutrition 58(2): 247-261.

Eleftheriou D, Benetou V, Trichopoulou A, La Vecchia C, Bamia C. Mediterranean diet and its components in relation to all-cause mortality: meta- analysis. Br J Nutr. 2018 Nov;120(10):1081-1097.

Evans PL, Prior JA, Belcher J, Hay CA, Mallen CD, Roddy E. Gender-specific risk factors for gout: a systematic review of cohort studies. Adv Rheumatol. 2019 Jun 24;59(1):24.

Fallahzadeh H, Cheraghi M, Amoori N, Alaf M. Red meat intake and risk of non-Hodgkin lymphoma: a meta-analysis. Asian Pac J Cancer Prev. 2014;15(23):10421-5.

Fan M, Li Y, Wang C, Mao Z, Zhou W, Zhang L, Yang X, Cui S, Li L. Dietary Protein Consumption and the Risk of Type 2 Diabetes: A Dose-Response Meta-Analysis of Prospective Studies. Nutrients. 2019 Nov 15;11(11):2783.

Fang X, Wei J, He X, An P, Wang H, Jiang L, et al. Landscape of dietary factors associated with risk of gastric cancer: A systematic review and dose-response meta-analysis of prospective cohort studies. Eur J Cancer 2015 Dec;51(18):2820–32.

Fardet A, Boirie Y. Associations between food and beverage groups and major diet-related chronic diseases: an exhaustive review of pooled/meta-analyses and systematic reviews. Nutr Rev. 2014 Dec;72(12):741-62.

Fardet A, Druesne-Pecollo N, Touvier M, Latino-Martel P. Do alcoholic beverages, obesity and other nutritional factors modify the risk of familial colorectal cancer? A systematic review. Crit Rev Oncol Hematol. 2017 Nov;119:94-112.

Farvid MS, Stern MC, Norat T, Sasazuki S, Vineis P, Weijenberg MP, Wolk A, Wu K, Stewart BW, Cho E. Consumption of red and processed meat and breast cancer incidence: A systematic review and meta-analysis of prospective studies. Int J Cancer. 2018 Dec 1;143(11):2787-2799.

Feskens EJ, Sluik D, van Woudenbergh GJ . Meat consumption, diabetes, and its complications. Curr Diab Rep 2013; 13: 298–306. Akesson A, Andersen LF, Kristjánsdóttir AG, Roos E, Trolle E, Voutilainen E, Wirfält E. Health effects associated with foods characteristic of the Nordic diet: a systematic literature review. Food Nutr Res. 2013 Oct 9;57.

Fogelholm M, Anderssen S, Gunnarsdottir I, Lahti-Koski M. Dietary macronutrients and food consumption as determinants of long-term weight change in adult populations: a systematic literature review. Food Nutr Res. 2012;56.

Gao Y, Ma Y, Yu M, Li G, Chen Y, Li X, Chen X, Xie Y, Wang X. Poultry and Fish Intake and Pancreatic Cancer Risk: A Systematic Review and Meta-Analysis. Nutr Cancer. 2021 Jan 12:1-13.

Gathirua-Mwangi WG, Zhang J. Dietary factors and risk for advanced prostate cancer. Eur J Cancer Prev. 2014 Mar;23(2):96-109.

George ES, Sood S, Broughton A, Cogan G, Hickey M, Chan WS, Sudan S, Nicoll AJ. The Association between Diet and Hepatocellular Carcinoma: A Systematic Review. Nutrients. 2021 Jan 8;13(1):172.

Gnagnarella P, Caini S, Maisonneuve P, Gandini S. Carcinogenicity of High Consumption of Meat and Lung Cancer Risk Among Non-Smokers: A Comprehensive Meta-Analysis. Nutr Cancer. 2018 Jan;70(1):1-13.

Granic A, Dismore L, Hurst C, Robinson SM, Sayer AA. Myoprotective Whole Foods, Muscle Health and Sarcopenia: A Systematic Review of Observational and Intervention Studies in Older Adults. Nutrients. 2020 Jul 28;12(8):2257.

Grosso G, Micek A, Godos J, Pajak A, Sciacca S, Galvano F, Boffetta P. Health risk factors associated with meat, fruit and vegetable consumption in cohort studies: A comprehensive meta-analysis. PLoS One. 2017 Aug 29;12(8):e0183787.

Guo J, Wei W, Zhan L. Red and processed meat intake and risk of breast cancer: a meta-analysis of prospective studies. Breast Cancer Res Treat. 2015 May;151(1):191-8.

He K, Li Y, Guo X, Zhong L, Tang S. Food groups and the likelihood of non-alcoholic fatty liver disease: a systematic review and meta-analysis. Br J Nutr. 2020 Mar 6;124(1):1-13.

Hoorsan H, Mirmiran P, Chaichian S, Moradi Y, Akhlaghdoust M, Hoorsan R, Jesmi F. Diet And Risk Of Endometriosis: A Systematic Review Andmeta-Analysis Study. Iranian Red Crescent Medical Journal (Ircmj). 2017 [cited 2022February17];19(9):0-0. Available from: https://www.sid.ir/en/journal/ViewPaper.aspx?id=609149

Hou JK, Abraham B, El-Serag H. Dietary intake and risk of developing inflammatory bowel disease: a systematic review of the literature. Am J Gastroenterol. 2011 Apr;106(4):563-73.

Huang W, Han Y, Xu J, Zhu W, Li Z. Red and processed meat intake and risk of esophageal adenocarcinoma: a meta-analysis of observational studies. Cancer Causes Control. 2013 Jan;24(1):193-201.

Händel MN, Cardoso I, Rasmussen KM, Rohde JF, Jacobsen R, Nielsen SM, Christensen R, Heitmann BL. Processed meat intake and chronic disease morbidity and mortality: An overview of systematic reviews and meta-analyses. PLoS One. 2019 Oct 17;14(10):e0223883.

Händel MN, Rohde JF, Jacobsen R, Nielsen SM, Christensen R, Alexander DD, Frederiksen P, Heitmann BL. Processed meat intake and incidence of colorectal cancer: a systematic review and meta-analysis of prospective observational studies. Eur J Clin Nutr. 2020 Aug;74(8):1132-1148.

Iacoviello L, Bonaccio M, Cairella G, Catani MV, Costanzo S, D'Elia L, Giacco R, Rendina D, Sabino P, Savini I, Strazzullo P; Working Group for Nutrition and Stroke. Diet and primary prevention of stroke: Systematic review and dietary recommendations by the ad hoc Working Group of the Italian Society of Human Nutrition. Nutr Metab Cardiovasc Dis. 2018 Apr;28(4):309-334.

Jakobsen MU, Bysted A, Mejborn H, Stockmarr A, Trolle E. Intake of Unprocessed and Processed Meat and the Association with Cardiovascular Disease: An Overview of Systematic Reviews. Nutrients. 2021 Sep 22;13(10):3303.

Jiang G, Li B, Liao X, Zhong C. Poultry and fish intake and risk of esophageal cancer: A meta-analysis of observational studies. Asia Pac J Clin Oncol. 2016 Mar;12(1):e82-91.

Johnson CM, Wei C, Ensor JE, Smolenski DJ, Amos CI, Levin B, Berry DA. Meta-analyses of colorectal cancer risk factors. Cancer Causes Control. 2013 Jun;24(6):1207-22.

Johnson IT. The cancer risk related to meat and meat products. Br Med Bull. 2017 Jan 1;121(1):73-81.

Kaluza J, Wolk A, Larsson SC. Red meat consumption and risk of stroke: a meta-analysis of prospective studies. Stroke. 2012 Oct;43(10):2556-60.

Kazemi A, Barati-Boldaji R, Soltani S, Mohammadipoor N, Esmaeilinezhad Z, Clark CCT, Babajafari S, Akbarzadeh M. Intake of Various Food Groups and Risk of Breast Cancer: A Systematic Review and Dose-Response Meta-Analysis of Prospective Studies. Adv Nutr. 2021 Jun 1;12(3):809-849.

Kim K, Hyeon J, Lee SA, Kwon SO, Lee H, Keum N, Lee JK, Park SM. Role of Total, Red, Processed, and White Meat Consumption in Stroke Incidence and Mortality: A Systematic Review and Meta-Analysis of Prospective Cohort Studies. J Am Heart Assoc. 2017 Aug 30;6(9):e005983.

Kim SR, Kim K, Lee SA, Kwon SO, Lee JK, Keum N, Park SM. Effect of Red, Processed, and White Meat Consumption on the Risk of Gastric Cancer: An Overall and Dose⁻Response Meta-Analysis. Nutrients. 2019 Apr 11;11(4):826.

Kim Y, Je Y. Meat Consumption and Risk of Metabolic Syndrome: Results from the Korean Population and a Meta-Analysis of Observational Studies. Nutrients. 2018 Mar 22;10(4):390.

Koutsokera A, Kiagia M, Saif MW, Souliotis K, Syrigos KN. Nutrition habits, physical activity, and lung cancer: an authoritative review. Clin Lung Cancer. 2013 Jul;14(4):342-50.

Kouvari M, Notara V, Kalogeropoulos N, Panagiotakos DB. Diabetes mellitus associated with processed and unprocessed red meat: an overview. Int J Food Sci Nutr. 2016 Nov;67(7):735-43.

Kwok CS, Gulati M, Michos ED, Potts J, Wu P, Watson L, Loke YK, Mallen C, Mamas MA. Dietary components and risk of cardiovascular disease and all-cause mortality: a review of evidence from meta-analyses. Eur J Prev Cardiol. 2019 Sep;26(13):1415-1429.

Larsson SC, Wolk A. Red and processed meat consumption and risk of pancreatic cancer: meta-analysis of prospective studies. Br J Cancer. 2012 Jan 31;106(3):603-7.

Larsson SC, Orsini N. Red meat and processed meat consumption and all-cause mortality: a meta-analysis. Am J Epidemiol. 2014 Feb 1;179(3):282-9.

Latino-Martel P, Cottet V, Druesne-Pecollo N, Pierre FH, Touillaud M, Touvier M, Vasson MP, Deschasaux M, Le Merdy J, Barrandon E, Ancellin R. Alcoholic beverages, obesity, physical activity and other nutritional factors, and cancer risk: A review of the evidence. Crit Rev Oncol Hematol. 2016 Mar;99:308-23.

Li L, Ying XJ, Sun TT, Yi K, Tian HL, Sun R, et al. Overview of methodological quality of systematic reviews about gastric cancer risk and protective factors. Asian Pac J Cancer Prev 2012;13(5):2069–79.

Lippi G, Mattiuzzi C, Cervellin G. Meat consumption and cancer risk: a critical review of published meta-analyses. Crit Rev Oncol Hematol. 2016 Jan;97:1-14.

Lippi G, Mattiuzzi C, Sanchis-Gomar F. Red meat consumption and ischemic heart disease. A systematic literature review. Meat Sci. 2015 Oct;108:32-6.

Luo J, Yang Y, Liu J, Lu K, Tang Z, Liu P, Liu L, Zhu Y. Systematic review with meta-analysis: meat consumption and the risk of hepatocellular carcinoma. Aliment Pharmacol Ther. 2014 May;39(9):913-22.

Lupoli R, Vitale M, Calabrese I, Giosuè A, Riccardi G, Vaccaro O. White Meat Consumption, All-Cause Mortality, and Cardiovascular Events: A Meta-Analysis of Prospective Cohort Studies. Nutrients. 2021 Feb 20;13(2):676.

Mandair D, Rossi RE, Pericleous M, Whyand T, Caplin ME. Prostate cancer and the influence of dietary factors and supplements: a systematic review. Nutr Metab (Lond). 2014 Jun 16;11:30.

Micha R, Michas G, Mozaffarian D. Unprocessed red and processed meats and risk of coronary artery disease and type 2 diabetes--an updated review of the evidence. Curr Atheroscler Rep. 2012 Dec;14(6):515-24.

Mohammadi H, Jayedi A, Ghaedi E, Golbidi D, Shab-Bidar S. Dietary poultry intake and the risk of stroke: A dose-response meta-analysis of prospective cohort studies. Clin Nutr ESPEN. 2018 Feb;23:25-33.

Mourouti N, Kontogianni MD, Papavagelis C, Panagiotakos DB. Diet and breast cancer: a systematic review. Int J Food Sci Nutr. 2015 Feb;66(1):1-42. Abete I, Romaguera D, Vieira AR, Lopez de Munain A, Norat T. Association between total, processed, red and white meat consumption and all-cause, CVD and IHD mortality: a meta-analysis of cohort studies. Br J Nutr. 2014 Sep 14;112(5):762-75.

Neuenschwander M, Ballon A, Weber KS, Norat T, Aune D, Schwingshackl L, Schlesinger S. Role of diet in type 2 diabetes incidence: umbrella review of meta-analyses of prospective observational studies. BMJ. 2019 Jul 3;366:l2368.

Nowson CA, Service C, Appleton J, Grieger JA. The Impact of Dietary Factors on Indices of Chronic Disease in Older People: A Systematic Review. J Nutr Health Aging. 2018;22(2):282-296.

Nucci D, Fatigoni C, Amerio A, Odone A, Gianfredi V. Red and Processed Meat Consumption and Risk of Depression: A Systematic Review and Meta-Analysis. Int J Environ Res Public Health. 2020 Sep 14;17(18):6686.

O'Sullivan TA, Hafekost K, Mitrou F, Lawrence D. Food sources of saturated fat and the association with mortality: a meta-analysis. Am J Public Health 2013 Sep;103(9):e31–e42.

Paluszkiewicz P, Smolińska K, Dębińska I, Turski WA. Main dietary compounds and pancreatic cancer risk. The quantitative analysis of case-control and cohort studies. Cancer Epidemiol. 2012 Feb;36(1):60-7.

Papier K, Knuppel A, Syam N, Jebb SA, Key TJ. Meat consumption and risk of ischemic heart disease: A systematic review and meta-analysis. Crit Rev Food Sci Nutr. 2021 Jul 20:1-12.

Papp RE, Hasenegger V, Ekmekcioglu C, Schwingshackl L. Association of poultry consumption with cardiovascular diseases and all-cause mortality: a systematic review and dose response meta-analysis of prospective cohort studies. Crit Rev Food Sci Nutr. 2021 Sep 20:1-22.

Qu X, Ben Q, Jiang Y. Consumption of red and processed meat and risk for esophageal squamous cell carcinoma based on a meta-analysis. Ann Epidemiol. 2013 Dec;23(12):762-770.e1.

Quach P, El SR, Gomes J, Krewksi D. A systematic review of the risk factors associated with the onset and progression of primary brain tumours. Neurotoxicology 2016 May 17.

Rezaianzadeh, A., Ghorbani, M., Rezaeian, S., Kassani, A. Red Meat Consumption and Breast Cancer Risk in Premenopausal Women: A Systematic Review and Meta-Analysis. Middle East Journal of Cancer, 2018; 9(1): 5-12.

Rouhani MH, Salehi-Abargouei A, Surkan PJ, Azadbakht L. Is there a relationship between red or processed meat intake and obesity? A systematic review and meta-analysis of observational studies. Obes Rev. 2014 Sep;15(9):740-8.

Salehi M, Moradi-Lakeh M, Salehi MH, Nojomi M, Kolahdooz F. Meat, fish, and esophageal cancer risk: a systematic review and dose-response meta-analysis. Nutr Rev. 2013 May;71(5):257-67.

Salem AA, Mackenzie GG. Pancreatic cancer: A critical review of dietary risk. Nutr Res. 2018 Apr;52:1-13.

Saneei P, Willett W, Esmaillzadeh A. Red and processed meat consumption and risk of glioma in adults: A systematic review and meta-analysis of observational studies. J Res Med Sci 2015 Jun;20(6):602–12.

Shareghfarid E, Sangsefidi ZS, Salehi-Abargouei A, Hosseinzadeh M. Empirically derived dietary patterns and food groups intake in relation with Attention Deficit/Hyperactivity Disorder (ADHD): A systematic review and meta-analysis. Clin Nutr ESPEN. 2020 Apr;36:28-35.

Shi Y, Yu PW, Zeng DZ. Dose-response meta-analysis of poultry intake and colorectal cancer incidence and mortality. Eur J Nutr. 2015 Mar;54(2):243-50.

Shin J, Millstine D, Ruddy B, Wallace M, Fields H. Effect of Plant- and Animal-Based Foods on Prostate Cancer Risk. J Am Osteopath Assoc. 2019 Oct 21.

Singh JA, Reddy SG, Kundukulam J. Risk factors for gout and prevention: a systematic review of the literature. Curr Opin Rheumatol. 2011 Mar;23(2):192-202.

Solfrizzi V, Custodero C, Lozupone M, Imbimbo BP, Valiani V, Agosti P, Schilardi A, D'Introno A, La Montagna M, Calvani M, Guerra V, Sardone R, Abbrescia DI, Bellomo A, Greco A, Daniele A, Seripa D, Logroscino G, Sabbá C, Panza F. Relationships of Dietary Patterns, Foods, and Micro- and Macronutrients with Alzheimer's Disease and Late-Life Cognitive Disorders: A Systematic Review. J Alzheimers Dis. 2017;59(3):815-849.

Song P, Lu M, Yin Q, Wu L, Zhang D, Fu B, Wang B, Zhao Q. Red meat consumption and stomach cancer risk: a meta-analysis. J Cancer Res Clin Oncol. 2014 Jun;140(6):979-92.

Toi PL, Anothaisintawee T, Chaikledkaew U, Briones JR, Reutrakul S, Thakkinstian A. Preventive Role of Diet Interventions and Dietary Factors in Type 2 Diabetes Mellitus: An Umbrella Review. Nutrients. 2020 Sep 6;12(9):2722.

Vahid F, Davoodi SH. Nutritional Factors Involved in the Etiology of Gastric Cancer: A Systematic Review. Nutr Cancer. 2021;73(3):376-390.

Wallin A, Orsini N, Wolk A. Red and processed meat consumption and risk of ovarian cancer: a dose-response meta-analysis of prospective studies. Br J Cancer. 2011 Mar 29;104(7):1196-201.

van Meer S, Leufkens AM, Bueno-de-Mesquita HB, van Duijnhoven FJ, van Oijen MG, Siersema PD. Role of dietary factors in survival and mortality in colorectal cancer: a systematic review. Nutr Rev. 2013 Sep;71(9):631-41.

Wang C, Jiang H. Meat intake and risk of bladder cancer: a meta-analysis. Med Oncol. 2012 Jun;29(2):848-55.

Wang X, Lin X, Ouyang YY, Liu J, Zhao G, Pan A, Hu FB. Red and processed meat consumption and mortality: dose-response meta-analysis of prospective cohort studies. Public Health Nutr. 2016 Apr;19(5):893-905.

Vargas AJ, Thompson PA. Diet and nutrient factors in colorectal cancer risk. Nutr Clin Pract. 2012 Oct;27(5):613-23.

Veettil SK, Wong TY, Loo YS, Playdon MC, Lai NM, Giovannucci EL, Chaiyakunapruk N. Role of Diet in Colorectal Cancer Incidence: Umbrella Review of Meta-analyses of Prospective Observational Studies. JAMA Netw Open. 2021 Feb 1;4(2):e2037341.

Wei Y, Zou D, Cao D, Xie P. Association between processed meat and red meat consumption and risk for glioma: a meta-analysis from 14 articles. Nutrition. 2015 Jan;31(1):45-50.

Vernooij RWM, Zeraatkar D, Han MA, El Dib R, Zworth M, Milio K, Sit D, Lee Y, Gomaa H, Valli C, Swierz MJ, Chang Y, Hanna SE, Brauer PM, Sievenpiper J, de Souza R, Alonso-Coello P, Bala MM, Guyatt GH, Johnston BC. Patterns of Red and Processed Meat Consumption and Risk for Cardiometabolic and Cancer Outcomes: A Systematic Review and Meta-analysis of Cohort Studies. Ann Intern Med. 2019 Nov 19;171(10):732-741.

Vieira AR, Abar L, Chan DSM, Vingeliene S, Polemiti E, Stevens C, Greenwood D, Norat T. Foods and beverages and colorectal cancer risk: a systematic review and meta-analysis of cohort studies, an update of the evidence of the WCRF-AICR Continuous Update Project. Ann Oncol. 2017 Aug 1;28(8):1788-1802.

Wolk A. Potential health hazards of eating red meat. J Intern Med. 2017 Feb;281(2):106-122.

Wu J, Zeng R, Huang J, Li X, Zhang J, Ho JC, Zheng Y. Dietary Protein Sources and Incidence of Breast Cancer: A Dose-Response Meta-Analysis of Prospective Studies. Nutrients. 2016 Nov 17;8(11):730.

Xu B, Sun J, Sun Y, Huang L, Tang Y, Yuan Y. No evidence of decreased risk of colorectal adenomas with white meat, poultry, and fish intake: a meta-analysis of observational studies. Ann Epidemiol. 2013 Apr;23(4):215-22.

Xu J, Yang XX, Wu YG, Li XY, Bai B. Meat consumption and risk of oral cavity and oropharynx cancer: a meta-analysis of observational studies. PLoS One. 2014 Apr 15;9(4):e95048.

Xue XJ, Gao Q, Qiao JH, Zhang J, Xu CP, Liu J . Red and processed meat consumption and the risk of lung cancer: a dose-response meta-analysis of 33 published studies. Int J. Clin Exp Med 2014; 7: 1542–1553.

Yang C, Pan L, Sun C, Xi Y, Wang L, Li D. Red Meat Consumption and the Risk of Stroke: A Dose-Response Meta-analysis of Prospective Cohort Studies. J Stroke Cerebrovasc Dis. 2016 May;25(5):1177-1186.

Yang L, Dong J, Jiang S, Shi W, Xu X, Huang H, You X, Liu H. Red and Processed Meat Consumption Increases Risk for Non-Hodgkin Lymphoma: A PRISMA-Compliant Meta-Analysis of Observational Studies. Medicine (Baltimore). 2015 Nov;94(45):e1729.

Yang WS, Wong MY, Vogtmann E, Tang RQ, Xie L, Yang YS, Wu QJ, Zhang W, Xiang YB. Meat consumption and risk of lung cancer: evidence from observational studies. Ann Oncol. 2012 Dec;23(12):3163-3170.

Yang X, Li Y, Wang C, Mao Z, Zhou W, Zhang L, Fan M, Cui S, Li L. Meat and fish intake and type 2 diabetes: Dose-response meta-analysis of prospective cohort studies. Diabetes Metab. 2020 Oct;46(5):345-352.

Yip CSC, Lam W, Fielding R. A summary of meat intakes and health burdens. Eur J Clin Nutr. 2018 Jan;72(1):18-29.

Zeraatkar D, Johnston BC, Bartoszko J, Cheung K, Bala MM, Valli C, Rabassa M, Sit D, Milio K, Sadeghirad B, Agarwal A, Zea AM, Lee Y, Han MA, Vernooij RWM, Alonso-Coello P, Guyatt GH, El Dib R. Effect of Lower Versus Higher Red Meat Intake on Cardiometabolic and Cancer Outcomes: A Systematic Review of Randomized Trials. Ann Intern Med. 2019 Nov 19;171(10):721-731.

Zhang H, Hardie L, Bawajeeh AO, Cade J. Meat Consumption, Cognitive Function and Disorders: A Systematic Review with Narrative Synthesis and Meta-Analysis. Nutrients. 2020 May 24;12(5):1528.

Zhang S, Wang Q, He J. Intake of red and processed meat and risk of renal cell carcinoma: a meta-analysis of observational studies. Oncotarget 2017 Sep 29;8(44):77942–56.

Zhao GH, Jiang S, Yan LH, Xie LF, Yang H, Wang Y. Is poultry intake associated with colorectal cancer? Int J Colorectal Dis. 2016 May;31(5):1087-1088.

Zhao Z, Feng Q, Yin Z, Shuang J, Bai B, Yu P, et al. Red and processed meat consumption and colorectal cancer risk: a systematic review and meta-analysis. Oncotarget 2017c Oct 10;8(47):83306–14.

Zhao Z, Wang F, Chen D, Zhang C. Red and processed meat consumption and esophageal cancer risk: a systematic review and meta-analysis. Clin Transl Oncol. 2020 Apr;22(4):532-545.

Zhao Z, Yin Z, Pu Z, Zhao Q. Association Between Consumption of Red and Processed Meat and Pancreatic Cancer Risk: A Systematic Review and Meta-analysis. Clin Gastroenterol Hepatol 2017a Apr;15(4):486–93.

Zhao Z, Yin Z, Zhao Q. Red and processed meat consumption and gastric cancer risk: a systematic review and meta-analysis. Oncotarget. 2017b May 2;8(18):30563-30575.

Zhu H, Yang X, Zhang C, Zhu C, Tao G, Zhao L, Tang S, Shu Z, Cai J, Dai S, Qin Q, Xu L, Cheng H, Sun X. Red and processed meat intake is associated with higher gastric cancer risk: a meta-analysis of epidemiological observational studies. PLoS One. 2013 Aug 14;8(8):e70955.

Zhu HC, Yang X, Xu LP, Zhao LJ, Tao GZ, Zhang C, Qin Q, Cai J, Ma JX, Mao WD, Zhang XZ, Cheng HY, Sun XC. Meat consumption is associated with esophageal cancer risk in a meat- and cancer-histological-type dependent manner. Dig Dis Sci. 2014 Mar;59(3):664-73.

Zumel-Marne A, Castano-Vinyals G, Kundi M, Alguacil J, Cardis E. Environmental Factors and the Risk of Brain Tumours in Young People: A Systematic Review. Neuroepidemiology. 2019;53(3-4):121-141.

Åkesson A, Andersen LF, Kristjánsdóttir AG, Roos E, Trolle E, Voutilainen E, Wirfält E. Health effects associated with foods characteristic of the Nordic diet: a systematic literature review. Food Nutr Res. 2013 Oct 9;57.
